# Comparison of the Cancer Gene Targeting and Biochemical Selectivities of All Targeted Kinase Inhibitors Approved for Clinical Use

**DOI:** 10.1371/journal.pone.0092146

**Published:** 2014-03-20

**Authors:** Joost C. M. Uitdehaag, Jeroen A. D. M. de Roos, Antoon M. van Doornmalen, Martine B. W. Prinsen, Jos de Man, Yoshinori Tanizawa, Yusuke Kawase, Kohichiro Yoshino, Rogier C. Buijsman, Guido J. R. Zaman

**Affiliations:** 1 Netherlands Translational Research Center B.V., Oss, The Netherlands; 2 Carna Biosciences Inc., Kobe, Japan; University of Colorado, School of Medicine, United States of America

## Abstract

The anti-proliferative activities of all twenty-five targeted kinase inhibitor drugs that are in clinical use were measured in two large assay panels: (1) a panel of proliferation assays of forty-four human cancer cell lines from diverse tumour tissue origins; and (2) a panel of more than 300 kinase enzyme activity assays. This study provides a head-on comparison of all kinase inhibitor drugs in use (status Nov. 2013), and for six of these drugs, the first kinome profiling data in the public domain. Correlation of drug activities with cancer gene mutations revealed novel drug sensitivity markers, suggesting that cancers dependent on mutant *CTNNB1* will respond to trametinib and other MEK inhibitors, and cancers dependent on *SMAD4* to small molecule EGFR inhibitor drugs. Comparison of cellular targeting efficacies reveals the most targeted inhibitors for EGFR, ABL1 and BRAF(V600E)-driven cell growth, and demonstrates that the best targeted agents combine high biochemical potency with good selectivity. For ABL1 inhibitors, we computationally deduce optimized kinase profiles for use in a next generation of drugs. Our study shows the power of combining biochemical and cellular profiling data in the evaluation of kinase inhibitor drug action.

## Introduction

**Figure 1:**
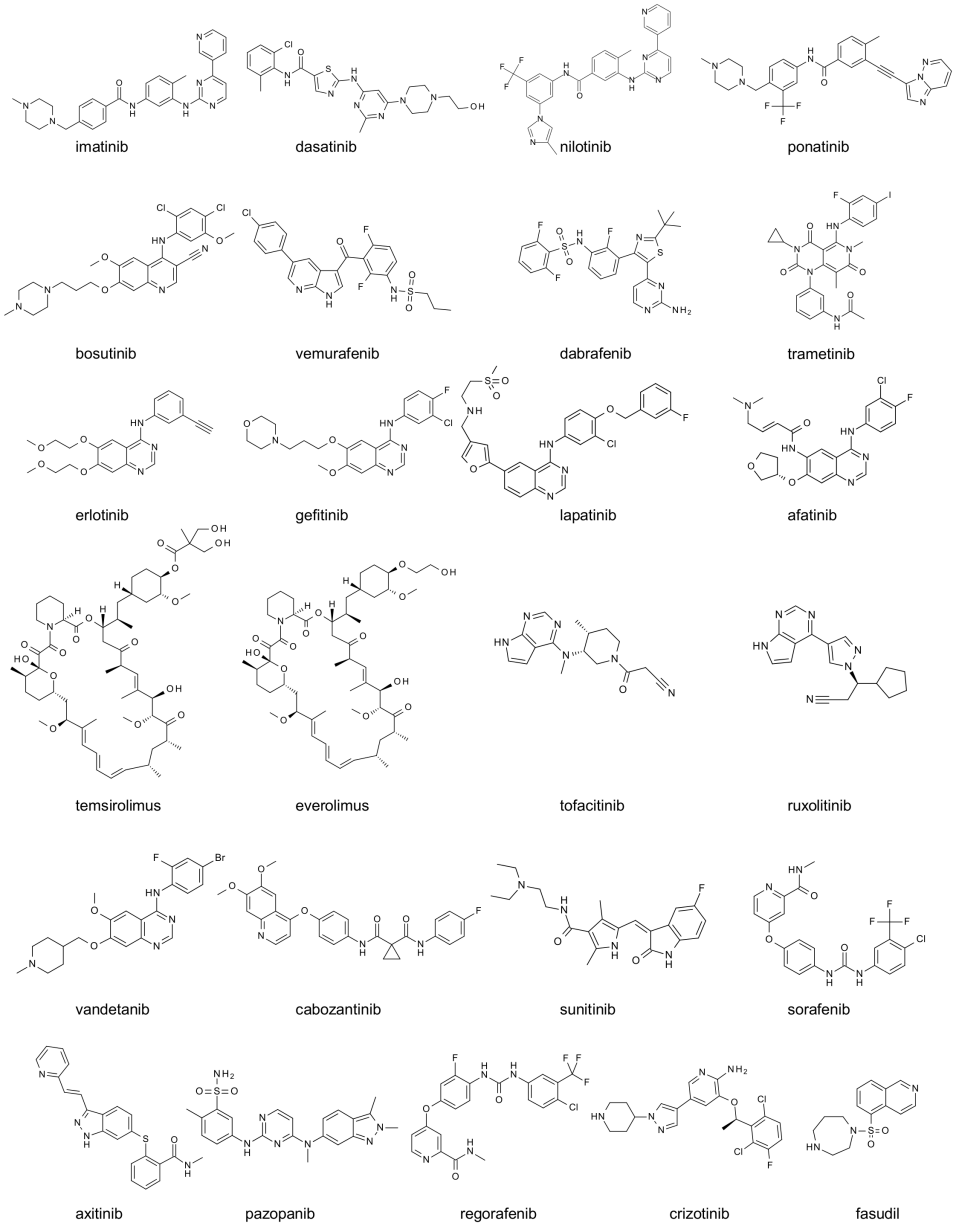
2D structures of the kinase inhibitors profiled in this study. All are kinase inhibitors that were approved for clinical use at Nov. 2013.

Targeted therapies significantly increase the efficiency of cancer therapy. They bring great benefit to patients because they improve survival rates with much less side effects than traditional cytotoxic therapies. Small molecule inhibitors of protein kinases are a prime example of the success of targeted therapy. There are currently (Nov. 2013) twenty-five kinase inhibitor drugs approved for clinical use, all except two for cancer (Table 1 and Figure 1). In 2012 protein kinases were the single most successful target class based on the number of approved new medicines by the U.S. Food and Drug Administration (FDA) and this trend continued in 2013 [1]. However, given the high attrition of drug candidates, the limited survival benefits of first generation therapies, the problem of drug resistance and the fact that targeted therapy is only of benefit to a small fraction of cancer patients, there is a need for novel and improved targeted kinase inhibitors.

**Table 1:** Overview of marketed kinase inhibitors, their applications and biochemical characteristics.

Inhibitor name	Marketed name	Clinical use^1^	Reported target	IC_50_ (nM) on relevant targets						Selectivity entropy^2^
**imatinib**	Gleevec	chronic myelogenous leukemia	ABL	ABL1	190	ABL1(T315I)	>10000			0.8
**dasatinib**	Sprycel			ABL1	0.27	ABL1(T315I)	1140			3.2
**nilotinib**	Tasigna			ABL1	18.5	ABL1(T315I)	9170			2.3
**ponatinib** 3	Iclusig			ABL1	2.7	ABL1(T315I)	6.4			*4*
**bosutinib**	Bosulif			ABL1	4.4	ABL1(T315I)	62			*3.1*
**vemurafenib**	Zelboraf	melanoma	BRAF	BRAF	41	BRAF(V600E)	520			*0.8*
**dabrafenib**	Tafinlar			BRAF	2.5	BRAF(V600E)	4.1			*2.5*
**trametinib**	Mekinist		MEK	MEK1	17	MEK2	42			*1.3*
**erlotinib**	Tarceva	non small cell lung cancer	EGFR	EGFR	0.75	HER2	>10000			0.5
**gefitinib**	Iressa			EGFR	0.51	HER2	3100			0.5
**lapatinib**	Tykerb			EGFR	4.9	HER2	9.8			1.0
**afatinib**	Gilotrif			EGFR	0.33	HER2	6.4			*0.9*
**temsirolimus**	Torisel	renal cell carcinoma	mTOR	mTOR^4^	∼2^4^					<0.1
**everolimus**	Afinitor			mTOR^4^	∼2^4^					<0.1
**tofacitinib**	Xeljanz	rheumatoid arthritis	JAKs	JAK2	2.1	JAK3	0.73			*0.9*
**ruxolitinib**	Jakafi	myelofibrosis		JAK2	0.32	JAK3	3.7			*1.1*
**vandetanib**	Caprelsa	medullary thyroid cancer	Growth factor kinases	VEGFR2	4.5	PDGFRα	9.1	RET	3.7	*2.7*
**cabozantinib**	Cometriq		(*i.e.* VEGFRs,	VEGFR2	2.5	MET	4	RET	11	*3.1*
**sunitinib**	Sutent	renal cell carcinoma	PDGFRs,	VEGFR2	20	PDGFRα	13	KIT	22.4	2.8
**sorafenib**	Nexavar		KIT)^1^	VEGFR2	29	PDGFRα	1	BRAF	292	1.6
**axitinib**	Inlyta			VEGFR2	1.2	VEGFR1	2	PDGFRα	3.5	*2.5*
**pazopanib**	Votrient			VEGFR2	7.1	PDGFRα	20	KIT	3.8	2.7
**regorafenib**	Stivarga	colorectal cancer		VEGFR2	2.6	PDGFRα	16	KIT	81	*2.5*
**crizotinib**	Xalkori	non small cell lung cancer	ALK	ALK	3.4	MET	7	ROS	2.3	*2.7*
**fasudil**	Eril	cerebral vasospasms	ROCK1/2	ROCK1	230	ROCK2	137			*3.4*

1 Based on FDA label information.

2 based on biochemical data presented in this work and in ref. [11]. Calculation of selectivity entropy as outlined in ref. [33]. Entropy numbers in italic were calculated from single concentration profilings.

3 ponatinib was recently withdrawn from US markets, but is still available outside the US.

4 temsirolimus and everolimus inhibit mTOR through complex formation with FKBP12, value indicates binding interaction with FKBP12 [55].

Crucial to the development of targeted therapies is the ability to couple drug response to a genetic marker such as mutation, translocation or overexpression of a cancer gene [2]. Despite that there are more than 500 kinases encoded by the human genome, current approved kinase inhibitor drugs act primarily through only about ten different targets (Table 1 and Table S1). Most kinase inhibitors for oncology act by inhibiting tumour cell proliferation, angiogenesis, or both [3]. Drug sensitivity biomarkers are therefore needed to support the development of new targeted therapies and to broaden the utility of marketed anti-cancer therapies.

**Figure 2:**
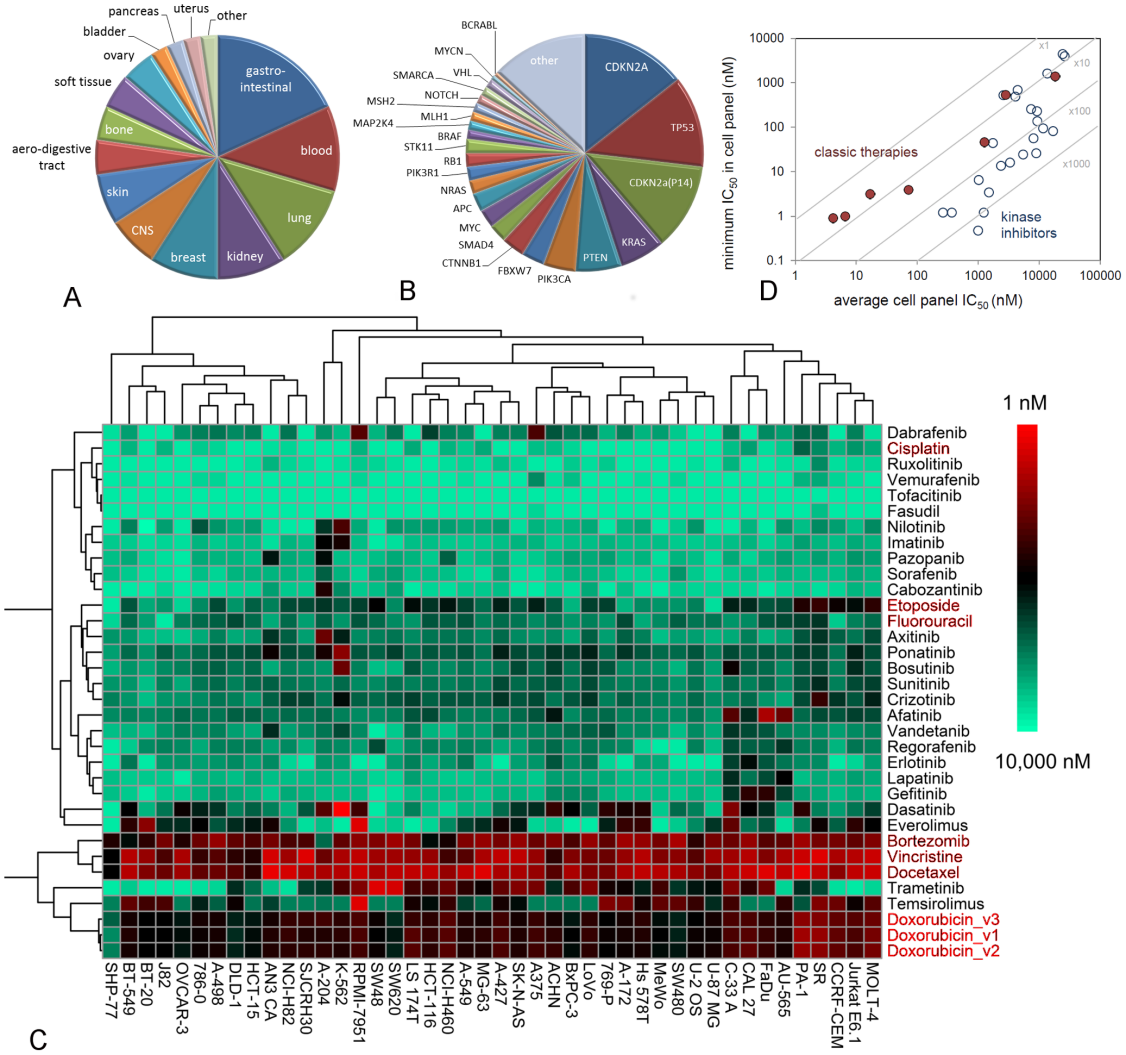
Cellular profiling of marketed kinase inhibitors. A: Tissue origin of cell lines in the Oncolines panel. B: Frequency of cancer gene changes in the cell panel, *i.e.*, mutations, translocations and copy number changes in the COSMIC Cell Line Project [4]. C: Hierarchical clustering of profiling data of marketed kinase inhibitor drugs in the 44-cell line panel. Unscaled ^10^logIC_50_s were used. Doxorubicin_123 is a triplicate profiling for control. Non-kinase inhibitors are coloured red. D: Kinase inhibitors have a greater selectivity in the cell panel than classic cytotoxic agents (5-fluorouracil, cisplatin, vincristine, doxorubicin, etoposide, docetaxel and bortezomib).

To better predict patient responder populations at an early stage in drug development, and to better understand kinase drug action, we have established a cancer cell line panel of forty-four cell lines that have been derived from a wide diversity of human tumours (Figure 2A). The cancer gene mutations that drive the cancerous phenotype of most of the cell lines have been characterized in the COSMIC Cell Lines (CCL) project [4]. Our panel contains representatives of all well-known oncogenes and tumour suppressors, that in a large cell panel sum up to more than 90% of all documented genetic changes (Table S2) [4]. Twenty-three of these frequent genetic changes occur in at least two cell lines (Figure 2B and Table S3).

Recent studies have demonstrated that cell line panels can be used to identify new markers of drug sensitivity by coupling drug response to the presence of cancer gene mutations [4]–[7]. These studies have used cell panels with up to 400–1000 cell lines, to discover novel sensitivities related to rare genetic variants. However, such panels are impractical for routine use [8]. Smaller panels are experimentally more accessible and can also give useful data, as has been demonstrated for the sixty cell line panel of the National Cancer Institute (NCI60), in which since the 1990s more than 300,000 compounds have been characterized [9], and the forty-five cell line panel of the Japanese research foundation [10].

To compare the anti-proliferative activities of all kinase inhibitor drugs that have been approved for clinical use, we have profiled them in our forty-four cell line panel. We correlated drug activities to cancer gene mutations and identified new drug sensitivity markers for MEK and EGFR inhibitors. In addition, the cell panel data were used to quantitatively compare the relative targeting efficacy of drugs designed to inhibit the same kinase.

To further study biochemical origins of differential effects in cellular targeting we profiled all kinase inhibitor drugs on a panel of enzyme activity assays of more than 300 wild-type and mutant kinases [11]. Whereas extensive biochemical selectivity data are available for many kinase inhibitors [12]–[14], this provides the first extensive profiles of the approved drugs cabozantinib [15], dabrafenib [16], ponatinib [17], regorafenib [18], trametinib [19] and vemurafenib [20] (Table 1). Combination of the cellular and biochemical datasets reveals that biochemical potency and selectivity are independent contributors to the efficient targeting of genetic drivers in tumour cells. In addition, specific off-target activities can positively contribute to targeting, as we show for ABL1 inhibitors, by computationally linking kinome profiles to cellular targeting. Our study shows that cell panel profiling in combination with biochemical panel profiling is a powerful tool in finding new applications for existing inhibitors, and the design of optimally targeted inhibitors.

## Results

### Composition and validation of the Cell Panel

A panel of forty-four human cancer cell lines was assembled from the American Type Culture Collection (ATCC). The cell lines were selected to represent both a wide range of different tissue tumour types (Figure 2A) and many different genetic alterations in oncogenes and tumour suppressor genes (Figure 2B). Public DNA sequence information from the CCL project [4] and the Cancer Cell Line Encyclopedia (CCLE) [5] were used to select the cell lines. For all cell lines, we developed proliferation assays using measurement of intracellular ATP content as an indirect readout of cell number. Compared to other cell panels, the panel (Oncolines) has increased genetic diversity (Figure S1) and the test concentrations span a wider range: nine points from 32 μM to 3.2 nM. We did not estimate compound activities by extrapolation outside the testing range, as was carried out in another study [4]. Instead, to ensure that all IC_50_s fell within the testing range, it was extended to subnanomolar concentrations in the case of very potent compounds. To preserve cell line characteristics, the cell lines were cultured in the media recommended by the original investigators who deposited the cell lines, and by ATCC. All cells used were within nine passages from the original ATCC vial.

The accuracy of cellular response data is getting increased attention [21]–[23]. By following a standardized workflow and implementing stringent quality criteria, we achieved a maximal IC_50_ shift of a factor 2 and a standard deviation of 0.07 on ^10^logIC_50_ values (a factor of 1.17), based on multiple independent measurements of the same compounds across the panel (Figure S2). To benchmark this value, we investigated reproducibility in public datasets. Despite the general consensus that public data sets of compound profiling experiments are of high value to the drug discovery community, information on the reproducibility of the data is sparse. For the NCI60 panel a maximum variation of IC_50_ of a factor 11 was observed when the same compound was measured on two different occasions (Figure S2C) Furthermore, in a recent analysis of chEMBL data [21], when two groups in different laboratories measured the same constant, a standard deviation of 0.8 in ^10^logIC_50_s was found. This translates to a factor of 10^0.8^ = 6 as standard deviation in IC_50_s. We therefore conclude that our cell line profiling data are highly reproducible.

To determine whether the panel has sufficient size, we performed a power analysis. Depending on the number of cell lines that carry a specific cancer gene mutation, an IC_50_ shift of 2 to 10 times between responders and non-responders is statistically significant (Table S4). These limits fall well within responses usually observed [4], and therefore a 44-cell line panel is suitably large to perform drug responder analyses.

### Profiling of Clinical Kinase Inhibitors in the Cell Panel

In a comparative drug sensitivity analysis, we profiled all twenty-five kinase inhibitors in clinical use on all forty-four cell lines, together with six classic cytotoxic agents and the proteasome inhibitor bortezomib (Figure 2C, Table S5). All kinase inhibitor drugs approved for oncology showed anti-proliferative activity on at least some of the cell lines. Only tofacitinib and fasudil, the two drugs that are approved for non-cancer indications (Table 1), showed no or very poor inhibitory activity.

Clustering of all cell proliferation data (Figure 2C) confirmed that cytotoxic agents have relatively undiscriminatory activity against all cell lines. The profile of the proteasome inhibitor bortezomib resembles that of cytotoxic agents, illustrating that inhibiting a well-defined target does not result in a targeted therapy when the target performs a general physiological function. Of all cell lines, SHP-77 was the least sensitive to doxorubicin, cisplatin, docetaxel, etoposide, vincristine and bortezomib (Figure 2C), which coincides with its expression of multiple multi-drug-resistance mechanisms [24]. HCT-15 and DLD-1 are different in karyotype but originate from the same patient [25]. Consistently, the profiles of the two cell lines cluster together. SW-620 and SW-480 also originate from the same patient but do not cluster together, primarily because SW-620, which is derived from a metastasis, is substantially more sensitive to the MEK inhibitor trametinib (Figure 2C).

Clear targeted effects are shown by kinase inhibitor drugs, as many inhibit the proliferation of only a few cell lines. Which lines depends on their mechanism of action. For example, the EGFR inhibitors lapatinib, erlotinib and gefitinib cluster together because they inhibit the same subset of cell lines, most notably AU-565, FaDu, CAL 27 and C-33A, which originate from a variety of tissues and have the common characteristic that they overexpress *EGFR* (Table S3). The ABL1 inhibitors imatinib and nilotinib cluster together because they selectively inhibit the cell lines A-204 and K-562 that are dependent on ABL1 for growth (Figure 2C). However, other kinase drugs inhibit the growth of multiple cell lines, such axitinib, ponatinib, bosutinib, sunitinib and crizotinib, which cluster together in the heat map (Figure 2C), the mTOR inhibitors temsirolimus and everolimus, and the MEK inhibitor trametinib (Figure 2C). To further analyse the cellular selectivity of kinase inhibitors, we compared the most potent cellular IC_50_ of a compound, as a measure of specific cellular activity, with the average IC_50_ in the full panel, as a measure of general cellular toxicity. Classic cytotoxic therapies and bortezomib show a 10-fold difference between the average IC_50_ in the cell panel and the most potent IC_50_ (Figure 2D). In contrast, most kinase inhibitors showed a 100-fold difference, and dasatinib even a more than 1000-fold difference (Figure 2D), demonstrating that kinase inhibitors indeed achieve an improved selectivity window in comparison to classic chemotherapeutic agents.

### Biochemical Profiling of Clinical Kinase Inhibitors

**Figure 3:**
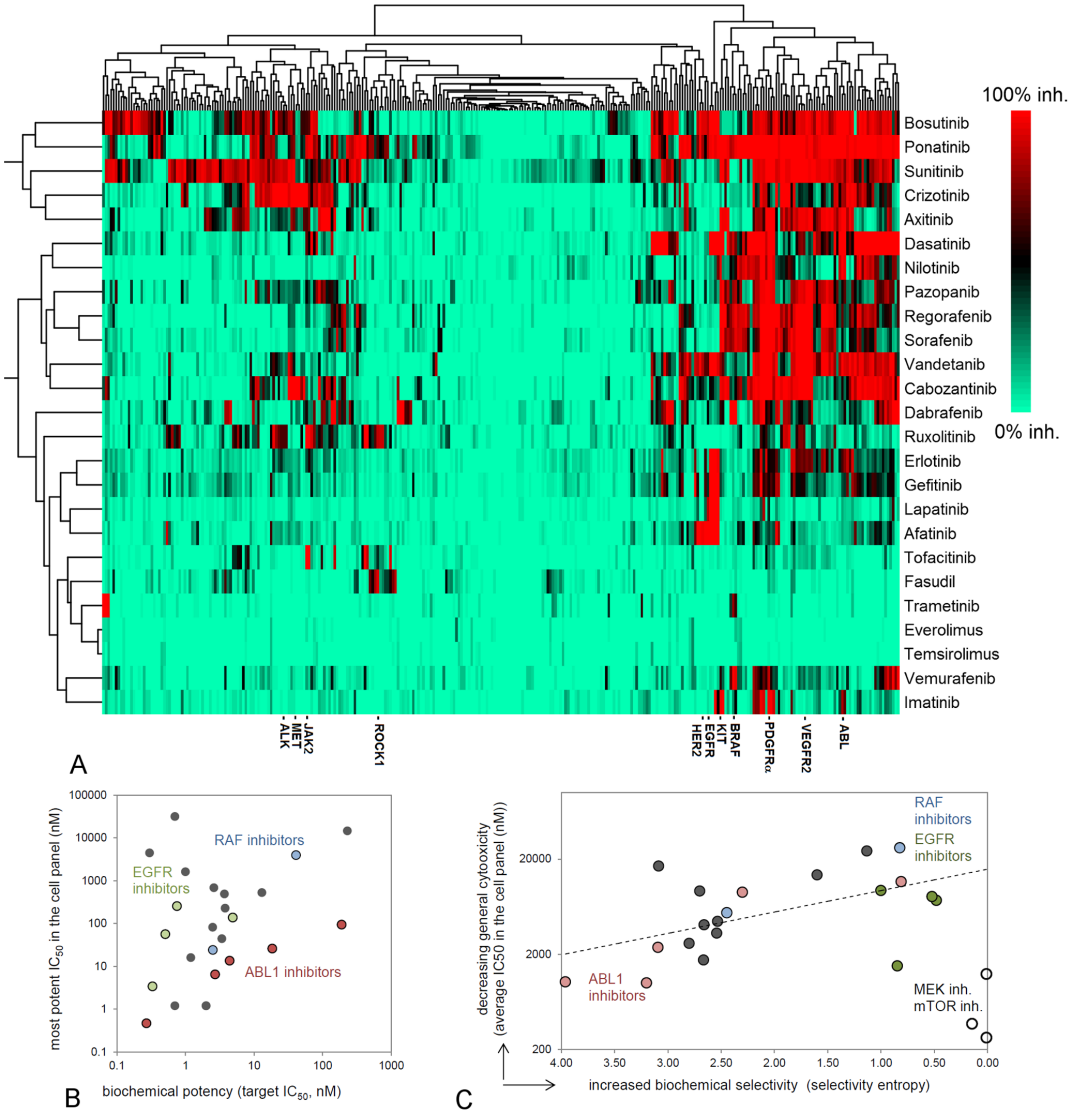
Biochemical profiling of marketed kinase inhibitors. A: Hierarchical clustering of inhibitory profiles of all kinase drugs in a panel of more than 300 biochemical kinase assays (%-inhibition at 1 μM inhibitor concentration). Trametinib, everolimus and temsirolimus show only minor inhibition, as mTOR and MEK kinase assays are not included in the panel. B: Potent biochemical IC_50_s on the biological target correlate with more potent cellular IC_50_s. C: Biochemical selectivity leads to a more selective response in the cell panel. Biochemical selectivity was quantified by selectivity entropy [33] and the selectivity of targeting cell growth was expressed by the average IC_50_ in the cell panel. Non-oncology drugs fasudil and tofacitinib were deleted from the analysis because of lack of response. Open circles: the mTOR and MEK inhibitors everolimus, temsirolimus and trametinib, respectively.

To relate the anti-proliferative activity of kinase inhibitor drugs to the inhibition of specific kinase targets, all compounds were profiled at a single concentration on a panel of more than 300 biochemical kinase assays (Figure 3A, Table S6) [11]. Additionally, for the most important targets, IC_50_ values were determined (Table 1). For vemurafenib, dabrafenib, trametinib, regorafenib and cabozantinib, this is the first large kinome profile in the public domain. A comparison of the approved RAF inhibitors vemurafenib and dabrafenib shows that dabrafenib is much more potent than vemurafenib on wild type BRAF and mutant BRAF(V600E). Dabrafenib also inhibits substantially more kinases (Table 1; Figure 3A). The first profile of trametinib reveals that, as most MEK inhibitors [26], it is exquisitely selective (Figure 3A). Regorafenib is a structural analog of sorafenib and shows a similar biochemical profile (Figure 3A). Regorafenib has been classified as more potent [18]. However, the data show that this is true for its inhibition of VEGFR2, a target of angiogenic drugs, but not for PDGFRα, a target in gastro-intestinal stromal tumours, an indication for which regorafenib is approved as well (Table 1). Biochemical inhibition of TIE2, another receptor involved in angiogenesis, was minor, consistent with a previous report (Table S6) [18]. Instead, regorafenib has substantial additional inhibitory activity on several oncogenic kinases, including the Ephrin receptors and p70S6K, that might contribute to its differential clinical profile [27]. Cabozantinib has been characterized as a combined VEGFR2, MET and RET inhibitor and is one of the most potent VEGFR2 inhibitors (Table 1). It is approved for use in medullary thyroid carcinoma, consistent with its potent inhibition of RET [28]. However, this is not a distinguishing characteristic of cabozantinib, as all growth factor kinase receptor inhibitors, and many ABL1 inhibitors are potent RET inhibitors (Table S6). Cabozantinib’s activity on MET, another important drug target [29], is much more special, as crizotinib is currently the only other approved drug that inhibits this kinase.

The biochemical profiles of all twenty-five kinase drugs in the same assay panel allow us to study how biochemical potency and selectivity influence general cellular targeting. This is important, as in the kinase field, selectivity of new drug candidates is a much-debated issue [30]–[32]. Improved biochemical potency correlates with improved cellular IC_50_s, and the strength of this relation is target-dependent (Figure 3B). To monitor biochemical selectivity, we summarized the kinome profiles by calculating the selectivity entropy (Table 1) [33]. The lower this value, the more selective a compound. A lower biochemical selectivity entropy is expected to result in less general cellular toxicity as determined by average cell panel IC_50_ and this is indeed the case for many inhibitors (Figure 3C). Axitinib, ponatinib, bosutinib, sunitinib and crizotinib have a high entropy (Table 1) and show broad cellular activity (Figure 3C). The cellular toxicity of EGFR, ABL1 and BRAF(V600E) inhibitors also improves with increasing selectivity (Figure 3C). The exceptions are the mTOR and MEK inhibitors which are biochemically highly selective inhibitors (Table 1, Figure 3A) but inhibit the proliferation of many cells. This confirms that MEK and mTOR drive the proliferation of many cell lines, and illustrates that the selectivity of a cellular response also depends on the biological target.

### Genetic Markers of Drug Sensitivity

To explore the biology underlying the cellular responses, we investigated the genetic determinants of response to the twenty-five kinase inhibitor drugs in an unbiased manner. We correlated any differences in IC_50_ by Anova analysis with mutations, translocations, mRNA overexpression and DNA copy number changes in a set of highly frequent and validated cancer genes (Table S3 and Figures S3 to S5). Several known associations of targeted therapies were used to validate the cell panel as an investigational tool for the discovery of new drug sensitivity markers. For instance, nutlin 3a, a compound stabilizing the interaction of p53 with MDM2, inhibited the proliferation of cell lines wild-type for *TP53* more potently than cell lines expressing mutant *TP53* (Figure S3). The BRAF inhibitors vemurafenib and dabrafenib preferentially inhibited the proliferation of cell lines containing the *BRAF(V600E)* mutation. ABL1 inhibitors and EGFR inhibitors preferentially inhibited cell lines that are dependent on *ABL1* and *EGFR* oncogenes, respectively (Figures S4 and S5).

**Figure 4:**
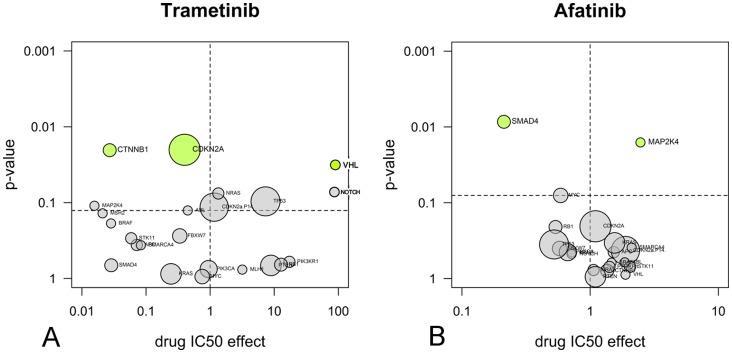
Anova analysis reveals novel drug response markers. A: the MEK inhibitor trametinib and B: the EGFR inhibitor afatinib. The volcano plots show the average IC_50_ shift between mutant and non-mutant cell lines (x-axis) and the significance from the Anova test (y-axis). Significance was corrected for multiple-testing and all associations above the threshold level (dotted line) are coloured green. Areas of circles are proportional with the number of cell lines carrying mutations.

With the Anova analysis, we discovered new drug sensitivity markers for MEK and EGFR inhibitors. The MEK inhibitor trametinib preferentially inhibited cell lines carrying mutations in *CTNNB1*, which encodes the transcription factor β-catenin (Figure 4A). The association was confirmed with two other MEK inhibitors, *i.e.* AZD6244 and PD0925301 (Figure S6). On average, the MEK inhibitors were between 12 and 37 times more potent in cell lines expressing mutant β-catenin in comparison to cell lines expressing only the wild-type protein. An additional interesting finding is that all four EGFR inhibitors, including afatinib, are more active in proliferation assays in cell lines harbouring a mutation in *SMAD4* (Figure 4B and Figure S5). The association was confirmed with two other EGFR inhibitors that are still in clinical development, *i.e.,* pelitinib and neratinib (Figure S7). The difference in the activity of the EGFR inhibitors in *SMAD4* mutant *versus* wild-type cells ranged from 2 to 12 times.

### Comparing Targeting Efficacy within Inhibitor Classes

**Figure 5:**
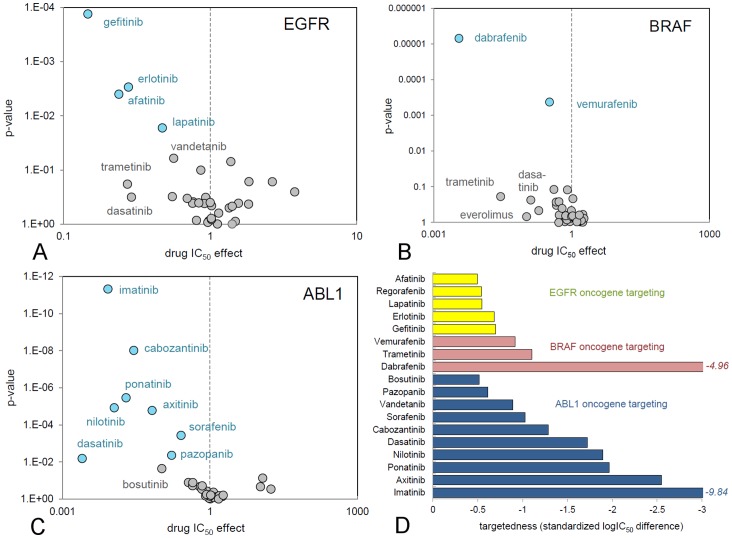
Comparison of the targeting efficacy of marketed inhibitors. Each circle represents a marketed kinase inhibitor and its targeted cell growth inhibition. A: Cell lines that overexpress *EGFR*. B: Cell lines containing the *BRAF(V600E)* mutation. C: Cell lines containing aberrant *ABL1* signalling. Compounds in the upper left corner of the plots have superior targeting. Statistically relevant associations after correction for multiple testing are coloured blue. D: Quantitative comparison of inhibitor targeting by standardization of IC_50_ shifts between sensitive and non-sensitive cell lines.

Analysis of the genetic determinants of cellular response allows comparison of the specificity of cellular targeting of different drugs that have been designed to inhibit the same molecular target, such as EGFR, ABL1, or BRAF inhibitors (Table 1, Figure 5).

EGFR inhibitors are one of the earliest examples of targeted therapies (Table 1). Gefitinib, erlotinib and afatinib have been approved for EGFR-overexpressing lung cancer. Also lapatinib has activity against EGFR (IC_50_, 4.9 nM, Table 1). These inhibitors are all highly selective (Table 1). In addition, the spectrum selective inhibitors vandetanib, bosutinib, ponatinib and dasatinib are potent EGFR inhibitors (Table S6). Overlay of the individual Anova analyses for EGFR shows that the selective inhibitors have a better correlation with *EGFR* expression levels and larger potency shifts than spectrum-selective inhibitors (Figure 5A). Also EGFR specific inhibitors, such as gefitinib and erlotinib, have a better targeting efficacy than the dually selective Her2/EGFR inhibitors lapatinib and afatinib, even though the irreversible inhibitor afatinib is most potent on EGFR. The most targeted EGFR inhibitor is gefitinib (most top-left in Figure 5A), which has similar biochemical properties as erlotinib (Table 1). Its superior targeting is probably related to specific off-target activities: *i.e.,* gefitinib is less active on ABL1 and more active on the EGFR(T290M) mutant and the Ephrin receptors, which can suppress EGFR by cross-talk [34].

The discovery that the growth of many tumours is driven by a specific mutation in the BRAF oncogene, *i.e., BRAF(V600E),* has led to the development of the RAF inhibitors vemurafenib and dabrafenib (Table 1). Trametinib is an inhibitor of MEK, which acts downstream of BRAF and is also registered for BRAF mutant cancers [19]. Sorafenib has been characterized as an inhibitor of BRAF [35], but has not been approved for BRAF-mutant cancers and poorly inhibits BRAF biochemically (Table 1). Anova analysis of the cell line profiling data reveals a strong association of the anti-proliferative activity of dabrafenib with mutant *BRAF(V600E)*, followed at a distance by vemurafenib (Figure 5B). Dabrafenib inhibited BRAF mutant cell lines with a 284 times lower IC_50_ than non-mutant cell lines. For vemurafenib the difference was 3-fold. There was no correlation between the cellular activity of sorafenib and *BRAF(V600E)*. Targeting efficacy of the RAF inhibitors is related to the enhanced biochemical potency of dabrafenib compared to vemurafenib, as dabrafenib is less selective (Table 1).

Another important class of kinase inhibitor drugs are those targeting ABL1, of which a re-arranged form, *i.e.,* BCR-ABL1, drives Philadelphia chromosome-positive chronic myelogenous leukemia (CML). Imatinib, nilotinib, dasatinib, ponatinib and bosutinib are approved drugs for this indication. However, also many growth factor kinase inhibitors such as crizotinib, vandetanib, axitinib and sunitinib are potent ABL1 inhibitors (Figure 3A, Table S6). Anova analysis of cancer cell line profiling data reveals a strong association with *ABL*-dependent cell growth for all CML-approved inhibitors, except bosutinib (Figure 5C). Dasatinib shows the most potent IC_50_ shift, which can be assigned to its superior potency. Because dasatinib is spectrum selective and inhibits the growth of many different cell lines, the significance (p-value) of the association is low. The most targeted ABL1 inhibitor in Figure 4C is actually the most selective ABL1 inhibitor, imatinib (Table 1).

### Quantification of Cancer Gene Targeting

To further compare inhibitors, we developed a quantitative measure of cancer gene targeting on the basis of cell panel data and response analysis. The average IC_50_ shift (ΔIC_50_) of a compound in the Anova analyses was taken as basis, as it indicates the difference in potency of a compound between sensitive (mutant) and insensitive (wild-type) cell lines. Another important parameter is the remaining variance between IC_50_s in the wild type or oncogene-carrying group of cell lines (σ*_mut_*_ or *wt*_), which is indicative of additional effects on cell growth besides the main inhibitor mechanism. To combine both values we selected the standardized mean difference (SMD) as a quantitative tool, which is calculated as ΔIC_50/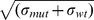
_. For the clinically used EGFR, ABL1 and BRAF kinase inhibitor drugs this quantity clearly shows that dabrafenib and imatinib are exceptionally targeted and that gefitinib and erlotinib are near-equivalent (Figure 5C), suggesting that the SMD of IC_50_s is a good tool to rank the targeting of drug candidates and existing therapies.

### Deducing Optimal Kinome Profiles

It has been argued that specific cross-reactivities, in addition to a primary biochemical activity, can positively contribute to the cellular targeting of kinase inhibitors by inhibiting resistance or feedback signalling [32], [36], [37]. Many research groups have tried to design specific, dual-activity, or even multiple-activity kinase inhibitors [31], [38], [39]. However, the question which inhibitor profile is most optimal to target a particular genetic driver has not been answered. Using cellular and biochemical data, we have started to derive such ‘optimal’ biochemical profiles for cellular targets.

**Figure 6:**
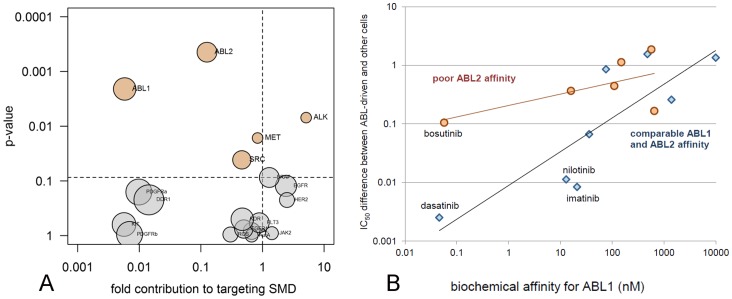
Determining the optimal kinome profile of an ABL inhibitor. A: Biochemical components of kinase inhibitors that contribute to the specific targeting of *ABL1* dependent cell growth. The circle labelled ABL1 refers to biochemical ABL1 inhibition. B: Inhibitors with equal ABL1 and ABL2 affinity in an independent dataset [12] are better in targeting *ABL1*-dependent cell growth than inhibitors with ABL1 activity alone. Poor ABL2 affinity signifies binding K_d_ differences between 4 and 26-fold compared to ABL1. Equal affinity signifies binding K_d_ differences between 0.5 and 4-fold.

Given the importance of ABL1 inhibitors (Table 1), we first searched for any biochemical activity, in addition to inhibition of ABL1, that may contribute to the targeting efficacy of this drug class. Twenty relevant kinases, including all targets of currently approved kinase inhibitor drugs, were selected as candidates that might confer beneficial secondary activities. If any of these kinases were inhibited by any of the 25 kinase inhibitor drugs (>80% in Table S6), the pair was labelled ‘active’, otherwise ‘inactive’. The resulting biochemical activity matrix was used in an Anova analysis together with the cellular targeting SMDs (Figure 5D) to identify biochemical activities that target cell lines that carry the *ABL1* oncogene. Aside from the expected identification of ABL1, this analysis surprisingly revealed ABL2 (ARG) as significant side-activity (Figure 6A). This finding was confirmed using elastic net regression analysis (not shown), and ABL2 was further validated by studying a separate dataset of binding K_d_s [12], confirming that ABL2-binding augments the targeting efficacy of ABL1 inhibitors in the cell line panel (Figure 6B). The contribution of ABL2 explains why bosutinib, which is a potent inhibitor of ABL1 but lacks ABL2 activity, has relatively weak targeting efficacy on *ABL1* oncogene-carrying cells compared to other ABL1 inhibitors (Figure 5C).

## Discussion

The development of selective kinase inhibitors has resulted in a number of breakthrough medicines for genetically well-defined patient populations [2], [40]. To support the development of the next generation of targeted kinase inhibitors, we have performed an in-depth analysis of the cellular and biochemical on-target efficacy of all kinase inhibitors in clinical use (Nov. 2013), by parallel profiling of all compounds on a panel of forty-four cell lines and a large kinase assay panel (Figures 2 and 3).

First, analysis of our data shows that there is potential for new applications of approved targeted kinase inhibitors (Figures S4 and S5). We discovered new drug sensitivity markers for MEK and EGFR inhibitors. MEK inhibitors were 12 to 37 times more active in cells harbouring mutated *CTNNB1* (Figure 4A). Although MEK inhibitors were included in the cell panel profiling studies of the Sanger Centre [4] and the Broad Institute [5], the association of MEK inhibition and *CTNNB1* was not observed in these studies. Studies in animal models and with patient derived material have shown that Wnt/β-catenin signalling stabilizes signalling via the RAS pathway, of which MEK is a component [41]. In lung smooth muscle cell line lines, the synthesis of β-catenin is regulated by MEK [42], suggesting that MEK inhibitors can stop aberrant Wnt signalling by inhibition of MEK-dependent β-catenin synthesis. Clinical treatment of *CTNNB1*-mutant cancers with MEK inhibitors is therefore worth further investigation.

Another new drug sensitivity marker was identified for EGFR inhibitors, which were not only active in *EGFR*-overexpressing cell lines, but also in cell lines harbouring *SMAD4* inactivating mutations (Figure 4B). The association is supported by a biological rationale, as many *EGFR* overexpressing cell lines also harbour inactivating mutations in *SMAD4* (*e.g.,* in our panel, BxPC3, CAL 27 and FaDu). Inactivation of *SMAD4* cooperates with KRAS to enhance EGFR expression levels [43]. In turn, this increases sensitivity to EGFR inhibitors [43], [44]. *SMAD4* mutation may therefore be further explored as a candidate marker for the selection of patients eligible for EGFR-inhibitor therapy.

Next our data shows that there is still potential to increase the targeting to highly validated cancer driving genes such as *BRAF*, *ABL1* and *EGFR*. Our data demonstrate that biochemical potency (illustrated by comparing dabrafenib to vemurafenib) and biochemical selectivity (illustrated by comparing the EGFR inhibitors) are both important for effective targeting of EGFR, BRAF and ABL1 inhibitors to *EGFR*, *BRAF(V600E)* and *ABL1* transformed cell lines (Figure 5). These insights give new directions for drug discovery. For instance, all second generation ABL1 inhibitors are less selective than the most targeted ABL1 inhibitor imatinib, because they are all optimized to inhibit the *ABL1(T315I)* resistance mutation (Table 1). A compound that is more selective (or more potent with equal selectivity) than imatinib is predicted to be even more targeted towards *ABL1*-driven cell growth. Along similar lines, a compound equipotent to dabrafenib but biochemically more selective is predicted to target *BRAF(V600E)* mutant melanoma more effectively *in vivo* than current available BRAF inhibiting drugs.

Finally, our combination of cell panel and biochemical panel data generate new insight in the relation between spectrum-selectivity of kinase inhibitors and their cellular targeting efficacy. Spectrum selectivity is generally not a beneficial property, unless specific and fortuitous combinations of biochemical activities are combined in one compound. We developed a novel method for *in silico* identification of such fortuitous polypharmacology. This revealed that ABL1 inhibitors benefit from a secondary ABL2 activity in the targeting of *ABL1*-driven cell growth (Figure 6). The association makes biological sense as *ABL2* is an oncogene in its own right [45] and *ABL1* and *ABL2* can have mutually supporting roles in the regulation of cell growth [46], [47]. We are currently expanding this analysis to more genetic drivers to provide ‘ideal’ kinome profiles for the design of multikinase inhibitors with improved targeting efficacy. Profiling methods, based on the combination of cellular and biochemical panels are therefore indispensable tools in the development of targeted therapy.

## Materials and Methods

### Cell Preparation

All cell lines were purchased from the American Type Culture Collection (ATCC, Manassas, VA, U.S.A.) and were authenticated by ATCC. Master and working cell banks and assay-ready stocks were prepared by subculturing in ATCC-recommended media and freezing according to ATCC recommended protocols. Master cell banks, working cell banks and assay stocks were prepared between 3, 6 and 9 passages from the original ATCC vial, respectively. A full list of the cell lines in the Oncolines panel is available in Table S7.

### Compound Preparation

All compounds were obtained from commercial suppliers (Table S1) and dissolved in 100% DMSO. At the day of the experiment, the compound stock was diluted in 3.16 fold steps in 100% DMSO to obtain a 9-point dilution series, followed by further dilution in aqueous buffer. A volume of 5 μl was transferred to the cells to generate a test concentration range from 3.16·10^−5 ^M to 3.16·10^−9^ M in duplicate. In case a compound showed low nanomolar or subnanomolar activity, it was further diluted 100 times and a new dose-response curve in duplicate was measured. The final DMSO concentration during incubation was 0.4% in all wells.

### Clinical Status of Compounds

Information on the therapeutic application of compounds was obtained from the label information of the US Food and Drug Administration (www.fda.gov).

### Cell Proliferation Assay

An assay stock was thawed and diluted in the media as recommended by ATCC, and dispensed in a 384-well plate, depending on the cell line used, at a concentration of 400–1600 cells per well in 45 μl medium. For each used cell line the optimal cell density was used and the number of cells per well was optimized to obtain a maximum assay window. Plated cells were incubated in a humidified atmosphere of 5% CO_2_ at 37°C. After 24 hours, 5 μl of compound dilution was added and plates were further incubated for another 72 hours after which 25 μl of ATPlite 1Step (PerkinElmer, Groningen, The Netherlands) solution was added to each well. Luminescence was recorded on an Envision multimode reader (PerkinElmer, Waltham, MA, U.S.A.).

### Controls

Firstly, the cell signal at the start of incubation was recorded separately in order to distinguish between cell population growth and cell death. Secondly, for each cell line, the maximum growth was measured by incubation of a duplicate without compound in the presence of 0.4% DMSO. Third, as a control for compound dilutions, the IC_50_ of the reference compound doxorubicin was measured on a separate plate. The IC_50_ was trended. If the IC_50_ was out of specification (*i.e*. <0.32 or >3.16 times deviating from historic average) the assay was invalidated. Fourth, the cellular doubling times of all cell lines were calculated from untreated wells. If the doubling time was out of specification (*i.e.,* <0.5 or >2.0 times deviating from historic average) the assay was invalidated.

### Curve Analysis

Percentage growth was used as the main y-axis signal. IC_50_s were fitted by non-linear regression using IDBS XLfit5 using a 4-parameter logistic curve, yielding a maximum signal, minimum signal, hill-parameter and IC_50_. In addition, using the initial cell counts, measures for cell death (LD_50_) and growth inhibition (GI_50_) were fitted [9]. All curves were checked manually. Furthermore, all curves were submitted to an F-test as implemented in XLfit5. If F values exceeded 1.5, curves were invalidated. For some compounds, biphasic curves were measured and in these cases the most potent effect was fitted. Curves were not extended to outside the measured range; all reported IC_50_s were measured within an enveloping concentration range.

### Clustering of Cellular and Biochemical Data

Cellular ^10^logIC_50_s were used as unscaled data to preserve their physical meaning and to compare absolute compound potencies. Biochemical %-inhibition data at 1 μM (see below) were restricted to values between 0% and 100% before clustering. For both datasets we applied unsupervised hierarchical clustering in the program R [48], using Ward’s minimum variance method [49], [50] with Euclidean distances for compounds and Spearman rank correlation [49] clustering for cell lines and kinases (Figure 2A and Figure 3A).

### Anova Analysis and Volcano Plots of Cell Line Mutations

Anova (analysis of variance) was used to test if there is a statistical correlation between a particular genetic change in the panel of cell lines and drug sensitivity. In principle, any number of genetic markers can be queried but because the number of hypotheses influences the reliability of associations in Anova analysis, we selected for our analysis the most frequent and most-studied oncogenes, as assessed from their frequency in a large cell line panel [4]. The genetic status (limited to ‘mutant’ or ‘wild-type’) of 23 frequently changed cancer genes was established for each cell line using public capillary sequencing data from the CCL database (Table S3) [4]. Cell lines K-562 and A-204 were labelled as having an *ABL1* transformation on basis of the literature [51], [52], as these cell lines were mislabelled as not *ABL1*-transformed in the CCL database. Mutations were treated as factors and the ^10^log IC_50_ as signal and analysed by n-way Anova in the statistical program R [48]. IC_50_ data were not normalized, but taken as-is, as this was giving good results for known associations. No interactions between factors were allowed. The minimum number of mutants for each gene to be incorporated in the test was 2, consistent with earlier analyses [4]. To minimize the effect of unbalanced sample populations, a type II sum-of-squares was used (type II Anova). Results of the Anova test were plotted as volcano plots, with at the y-axis the significance p-values and at the x-axis differences in the average ^10^log IC_50_ between mutant and wild-type cell lines. To correct for multiple testing, p-values were subjected to a Benjamini-Hochberg correction [53]. Genetic associations with a <20% false discovery rate were considered significant, unless otherwise indicated.

### Anova Analysis of Target Expression and Copy Number Data

For gene expression and copy number analysis, we limited the amount of hypotheses in Anova testing and reduced multiple-testing artefacts, by only considering the validated oncology targets EGFR, ERBB2, Kit, Met, PDGFRα and PDGFRβ (Table S3). Gene expression and copy number data for these targets were downloaded from the CCLE [5]. Any cell line with expression or copy number levels at 1 standard deviation above the average level seen for that gene in the 1037 cell lines of the CCLE was labelled as positive, the other cell lines as negative. Correlation between target overexpression and ^10^logIC_50_ was subsequently analysed with type II Anova as above.

### Kinase Profiling in a Biochemical Assay Panel

Previously, we described the profiling of nine kinase inhibitor drugs in a panel of more than 300 kinases using mobility shift assays and ELISA technology [11]. In the same panel and under the same conditions the other sixteen kinase inhibitor drugs were profiled. The compound concentration was 1 μM and the ATP concentration within two-fold of the K_M,ATP_ for every individual kinase (K_M_ bin). All 1 μM full profiles are given in Table S6. For the most important kinase targets for each compound, IC_50_s were determined (Table 1).

Selectivity entropy is a quantitative, single-value expression of the selectivity of compounds, allowing facile comparison of relative selectivity based on data from large pharmacological profiling experiments [14], [33]. Selectivity entropies were calculated from the IC_50_ data of Kitagawa *et al.* [11] or estimated from single concentration profiles as explained and validated in Figure S8 and Gubler *et al*. [54]. Only wild type kinases were included. Where applicable, estimated IC_50_s were replaced by measured IC_50_s to improve the selectivity entropy estimates (Table 1).

### Coupling of Biochemical Activity and Cellular Activity

Biochemical profiles were statistically linked to cellular profiles to deduce the optimal kinase profile for optimal cellular targeting activity. For the ABL1 oncogene, cellular targeting standardized mean differences (SMDs) were calculated for each kinase inhibitor drug as outlined above (Figure 5D). Each kinase drug was defined in terms of its biochemical inhibitor spectrum by assigning a label ‘active’ for each kinase that it inhibits >80% at 1 μM (Figure 3A), and ‘inactive’ for other kinases. The resulting matrix of inactive and active labels was subsequently used as explanatory variable in type II Anova analyses of the cellular targeting SMDs. Kinases considered in the definition of the biochemical inhibition spectra were the targets denoted in Table 1, supplemented with validated oncology targets picked from separate branches in the hierarchical clustering tree of kinases (Figure 3A). The final list comprised ABL1, ABL2, ALK, MET, ROS, EGFR, HER2, BRAF, KIT, PDGFRα, PDGFRβ, RET, JAK2, KDR, DDR1, AXL, AurA, SRC, FGFR1 and FLT3. Results were plotted in a volcano plot as outlined above, including Benjamini-Hochberg correction (Figure 6A) [53]. Kinase binding data of a subset of the clinically approved inhibitors [12] were used to validate the finding (Figure 6B).

## Supporting Information

Figure S1 **Characteristics of the CCL (COSMIC) panel and the NCI-60 panel.**
(DOCX)

Figure S2 **Reproducibility of the cell panel data.**
(DOCX)

Figure S3 **Volcano-analysis of nutlin 3a.**
(DOCX)

Figure S4 **Volcano-analysis of drug sensitivity of twenty-five approved kinase inhibitors and seven cytostatic therapies to twenty-three common genetic changes.**
(DOCX)

Figure S5 **Volcano-analysis of drug sensitivity of twenty-five approved kinase inhibitors and seven cytostatic therapies to genetic changes in growth factor signalling.**
(DOCX)

Figure S6 **Volcano-analysis of the MEK inhibitors PD-0325901 and selumetinib (AZD-6244).**
(DOCX)

Figure S7 **Volcano-analysis of the EGFR inhibitors pelitinib (EKB-569) and neratinib (HKI-272).**
(DOCX)

Figure S8 **Selectivity entropy (S_sel_) can be estimated on basis of single concentration data.**
(DOCX)

Table S1 **Names and sources of approved kinase inhibitor drugs used in this study.**
(DOCX)

Table S2 **Frequency of point mutations in validated cancer genes in the cell panel.**
(DOCX)

Table S3 **List of genes mutated in the Oncolines panel that have been used to investigate drug sensitivity.**
(DOCX)

Table S4 **Statistical power analysis to determine cutoff levels for significance in the cell line panel.**
(DOCX)

Table S5 **IC_50_s of 25 kinase inhibitors on 44 cell lines.**
(XLSX)

Table S6 **Biochemical inhibition of more than 300 kinases by 25 kinase inhibitors (%-inhibition values at 1 μM concentration).**
(XLSX)

Table S7 **List of all cell lines in the Oncolines panel.**
(DOCX)
