# Supplementary material for: Comparison of the Cancer Gene Targeting and Biochemical Selectivities of All Targeted Kinase Inhibitors Approved for Clinical Use
Source: PLoS One. 2014 Mar 20;9(3):e92146. doi: 10.1371/journal.pone.0092146 (PMC3961306; doi:10.1371/journal.pone.0092146)
Supplement: Figure S1 — Characteristics of the CCL (COSMIC) panel and the NCI-60 panel. (DOCX) [file pone.0092146.s001.docx]

Uitdehaag *et al*. supplementary Figure S1

**Figure S1**. Characteristics of the CCL (COSMIC) panel (639 cell lines) [4] and the NCI-60 panel [9]. Tissue origin of cell lines (top). Cancer gene mutations (bottom). Only point mutations (not translocations or copy number changes) are counted. The tissue diversity and genetic diversity of the CCL panel is represented well in the Oncolines panel (see Figure 2 in the main text).
