# Supplementary material for: Comparison of the Cancer Gene Targeting and Biochemical Selectivities of All Targeted Kinase Inhibitors Approved for Clinical Use
Source: PLoS One. 2014 Mar 20;9(3):e92146. doi: 10.1371/journal.pone.0092146 (PMC3961306; doi:10.1371/journal.pone.0092146)
Supplement: Figure S2 — Reproducibility of the cell panel data. (DOCX) [file pone.0092146.s002.docx]

Uitdehaag *et al*. supplementary Figure S2

**A
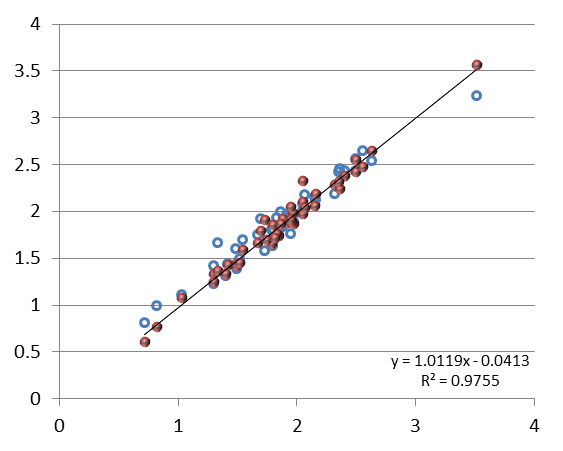
B**
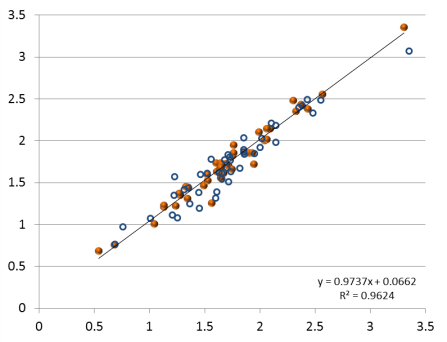
**C**
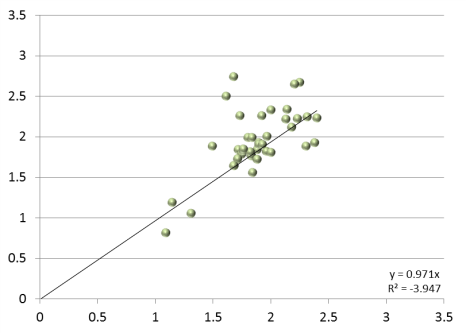


**Figure S2**. **Reproducability of the cell panel data.** A: Doxorubicin IC_50_ data from a panel profiling measured in three separate datasets, each in duplicate. Dataset 1 compared to 2 in red, closed circles. Dataset 1 compared to 3 in blue, open circles. B: GI_50_ data from the same triplicate. Every dot represents a separate cell line. The largest difference in IC_50_s and GI_50_s are log differences of 0.332 and 0.345, which are differences of 2.1- and 2.2-fold, respectively. The average standard deviation between ^10^logIC_50_s measured in the same cell line on separate occasions is 0.06 (a difference in IC_50_ of 1.14-fold). C: Doxorubicin GI_50_ data measured in the NCI60 panel at the Development Therapeutics Program [9] on two separate occasions in two concentration ranges, a rare example in which duplicate measurements are available for public data [21]. The largest GI_50_ difference is a factor of 11.
