# Supplementary material for: Comparison of the Cancer Gene Targeting and Biochemical Selectivities of All Targeted Kinase Inhibitors Approved for Clinical Use
Source: PLoS One. 2014 Mar 20;9(3):e92146. doi: 10.1371/journal.pone.0092146 (PMC3961306; doi:10.1371/journal.pone.0092146)
Supplement: Figure S3 — Volcano-analysis of nutlin 3a. (DOCX) [file pone.0092146.s003.docx]

Uitdehaag *et al*. supplementary Figure S3


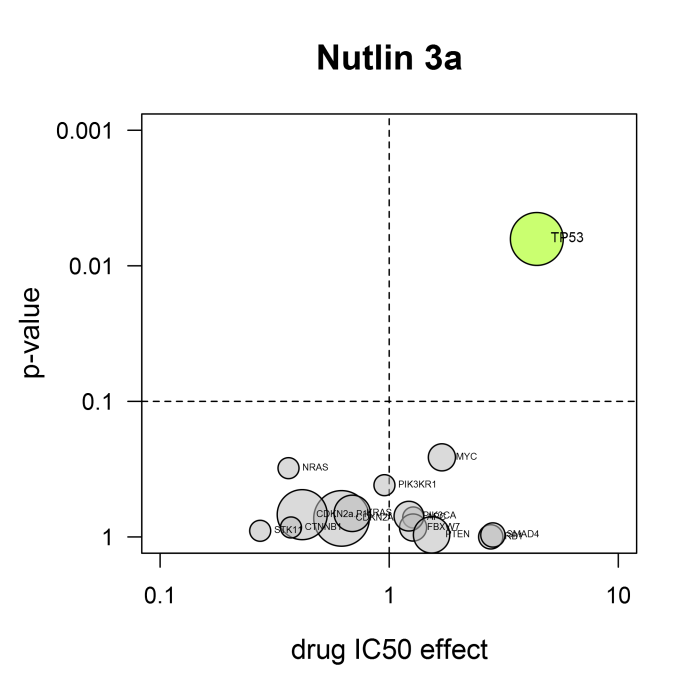


**Figure S3. Volcano-analysis of nutlin 3a**. Nutlin disturbs the interaction between MDM2 and p53 and works best in cell lines harbouring wild type p53 [4]. To bring out the effects clearly, cancer genes were analysed that occur at least three times in the panel, and the significance cutoff was set at a fixed level of 0.1.
