# Supplementary material for: Comparison of the Cancer Gene Targeting and Biochemical Selectivities of All Targeted Kinase Inhibitors Approved for Clinical Use
Source: PLoS One. 2014 Mar 20;9(3):e92146. doi: 10.1371/journal.pone.0092146 (PMC3961306; doi:10.1371/journal.pone.0092146)

Uitdehaag *et al.* supplementary Figure S5

**Figure S5**. **Volcano-analysis of drug sensitivity of twenty-five approved kinase inhibitors and seven cytostatic therapies to genetic changes in growth factor signalling.** Changes were analyzed in gene expression and DNA copy number of five receptors involved in growth factor signalling (CCLE data [5], for a list see Table S3). The p-value (y-axis in the vulcano plot) indicates the confidence level for association of particular receptor levels with a IC_50_ shift. The average factor of the IC_50_ shift is indicated on the x-axis. The areas of the circles are proportional to the number of mutants in the cell panel (each mutation is present at least twice). To compute significance, p-values were subjected to a Benjamini-Hochberg multiple testing correction [53], and only genetic associations with a <20% false discovery rate were colored green.

**Kinase inhibitors (alphabetical order)**


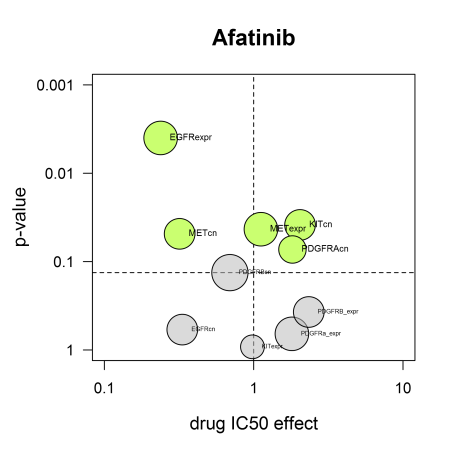

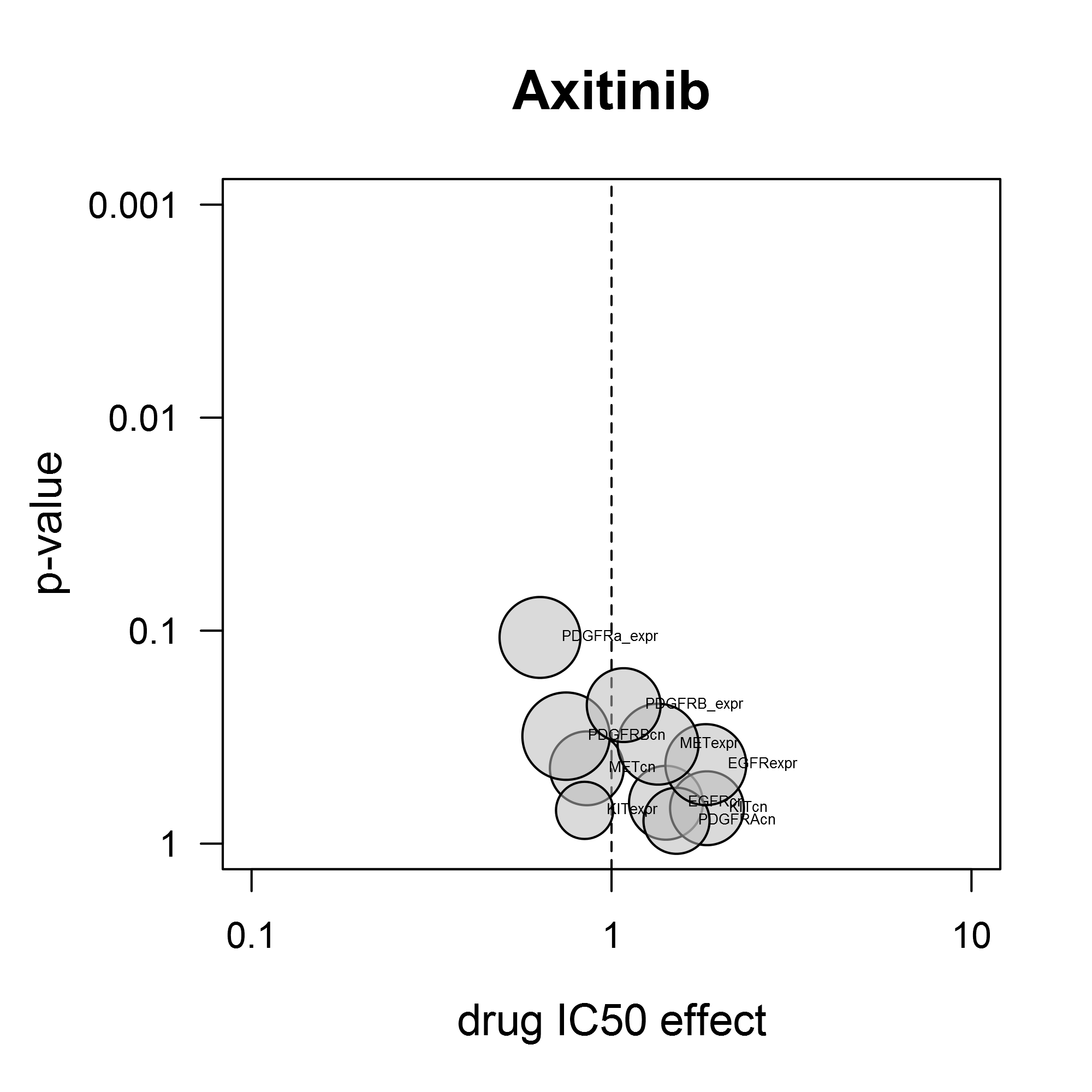

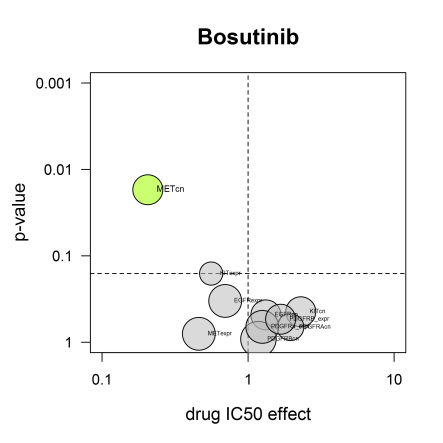


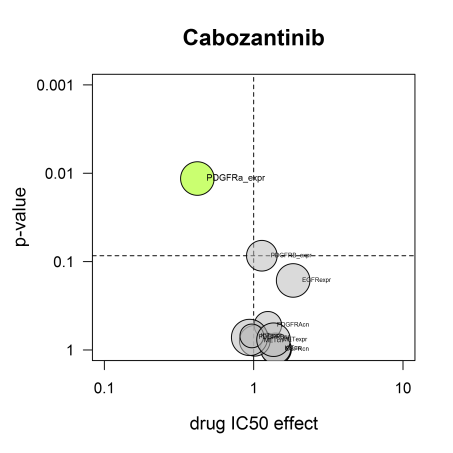

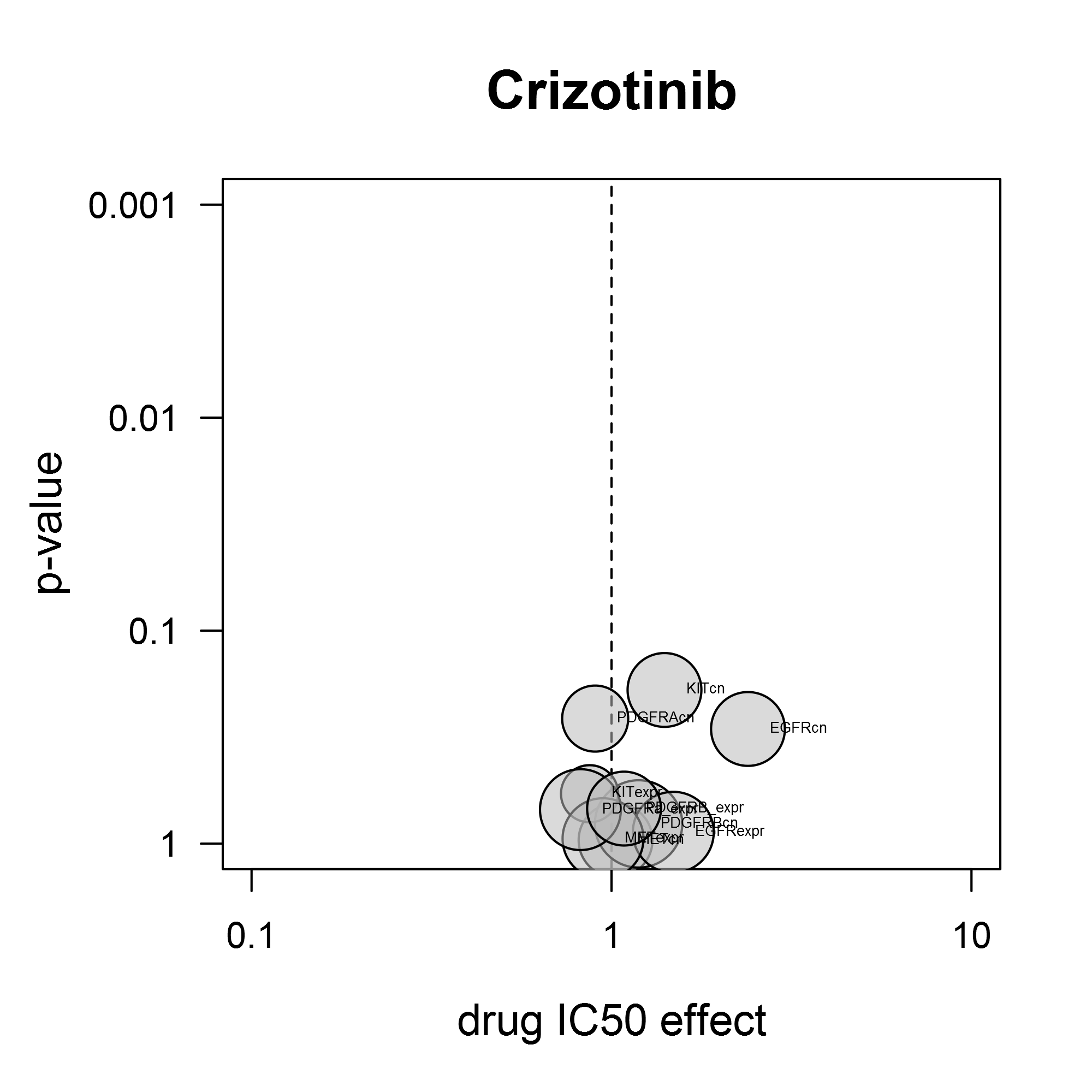

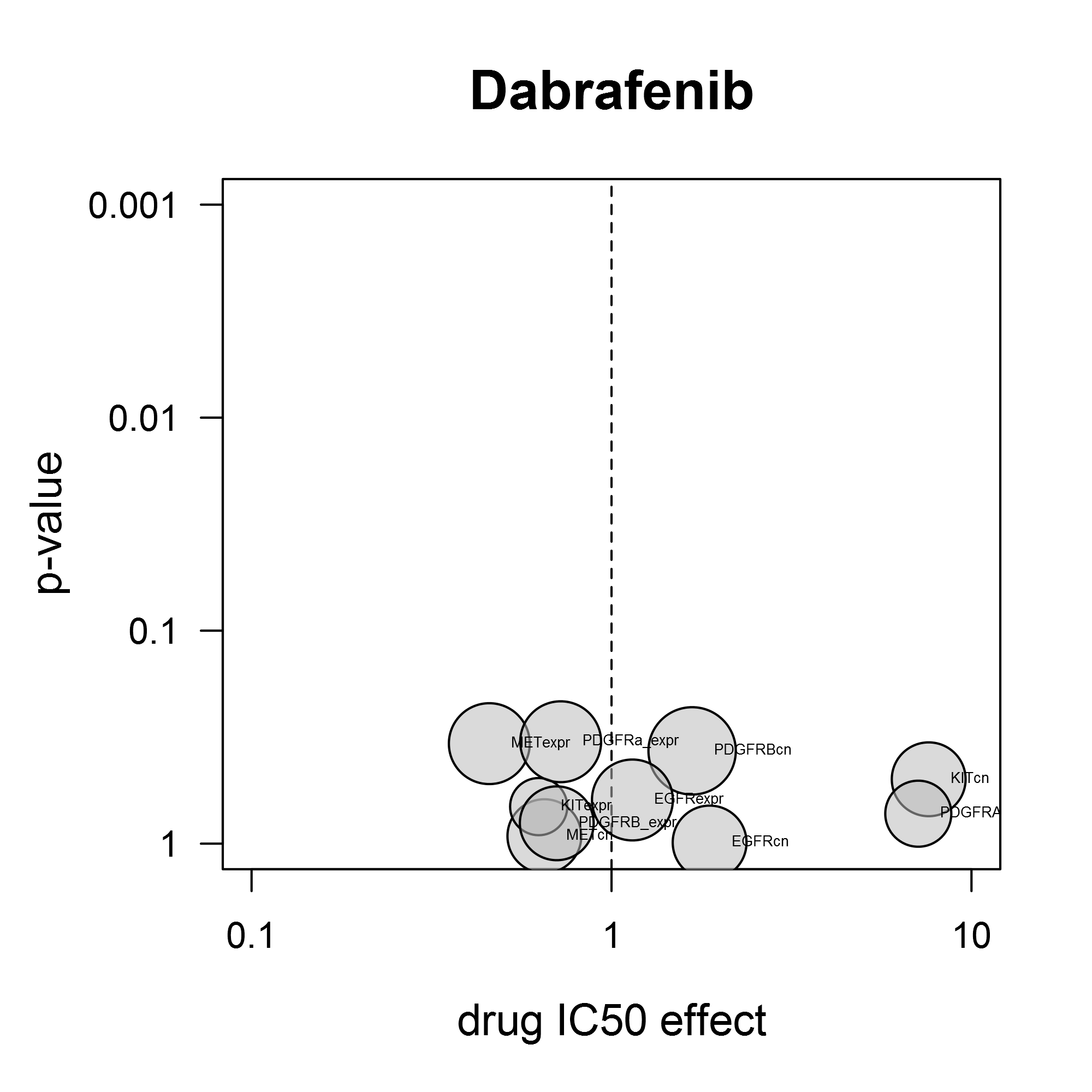


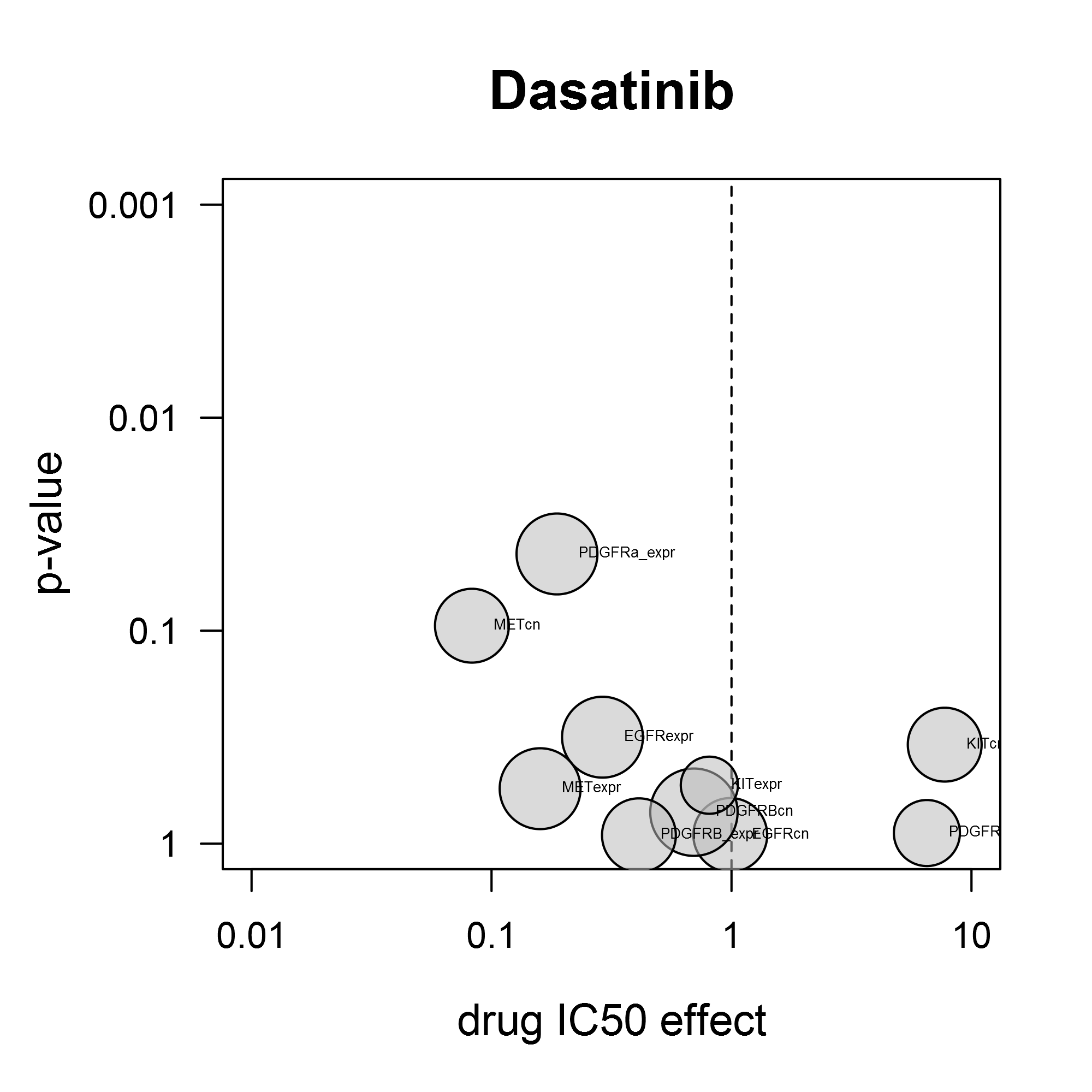

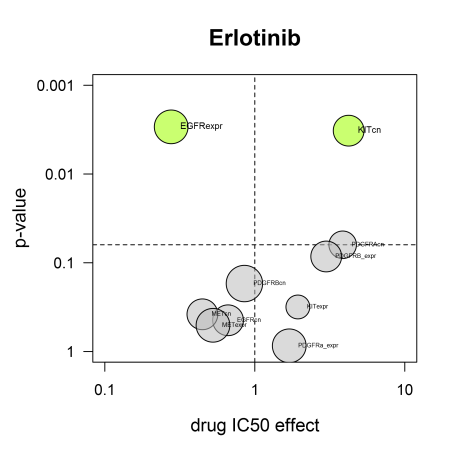

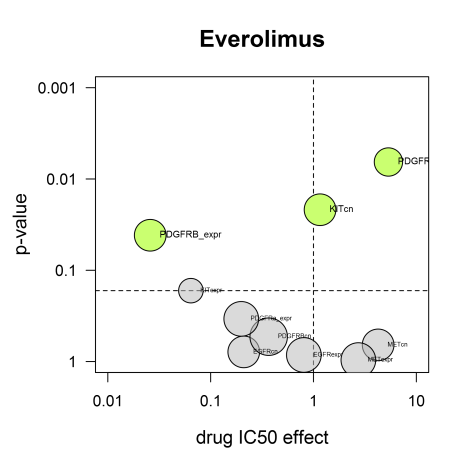


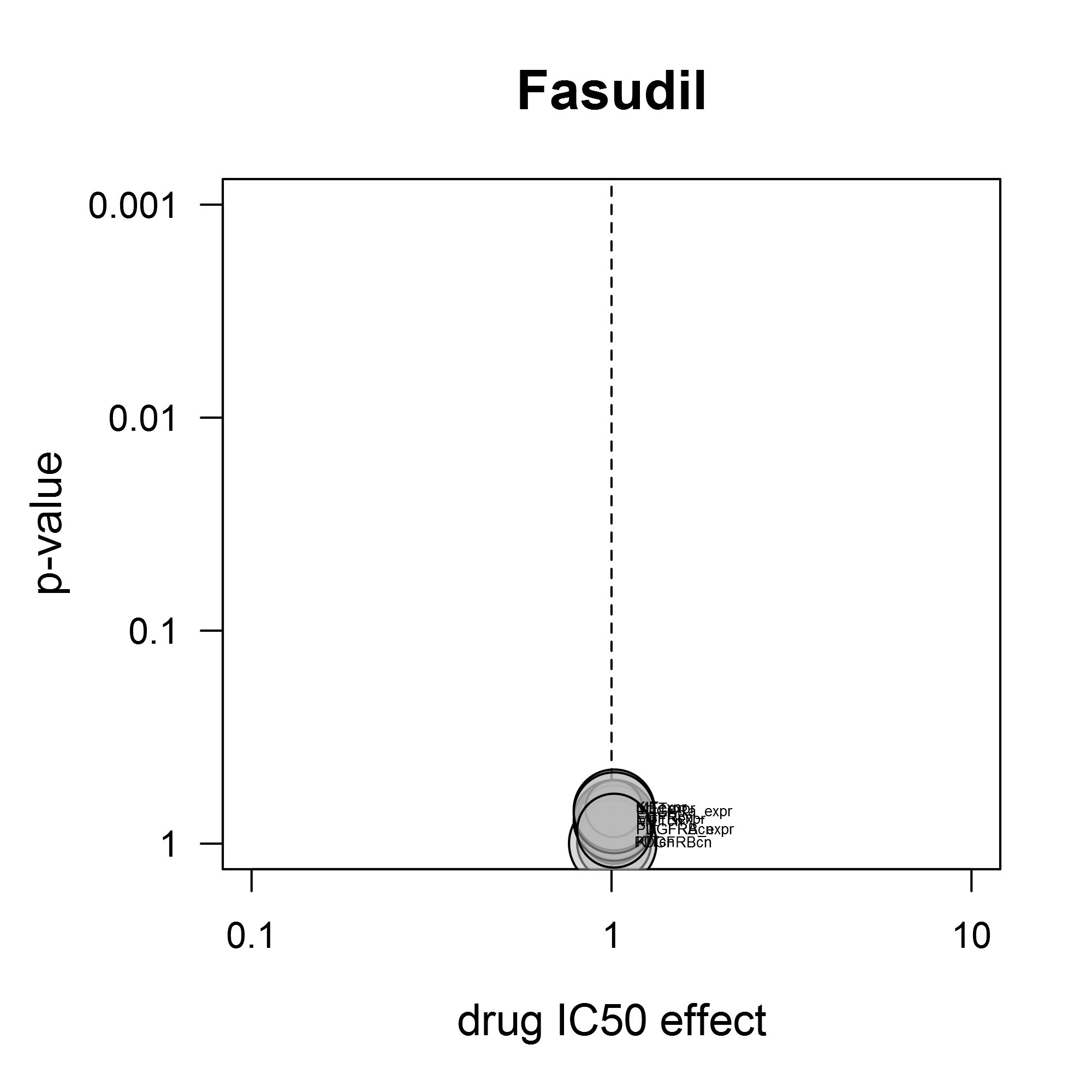

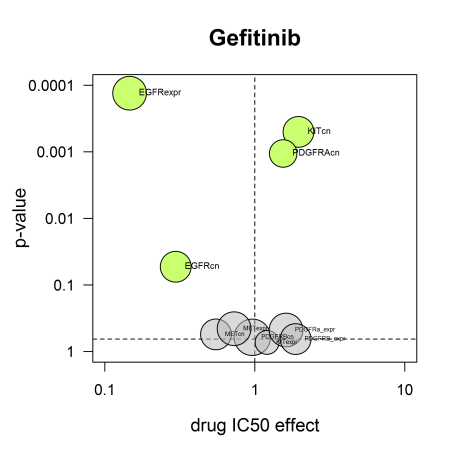

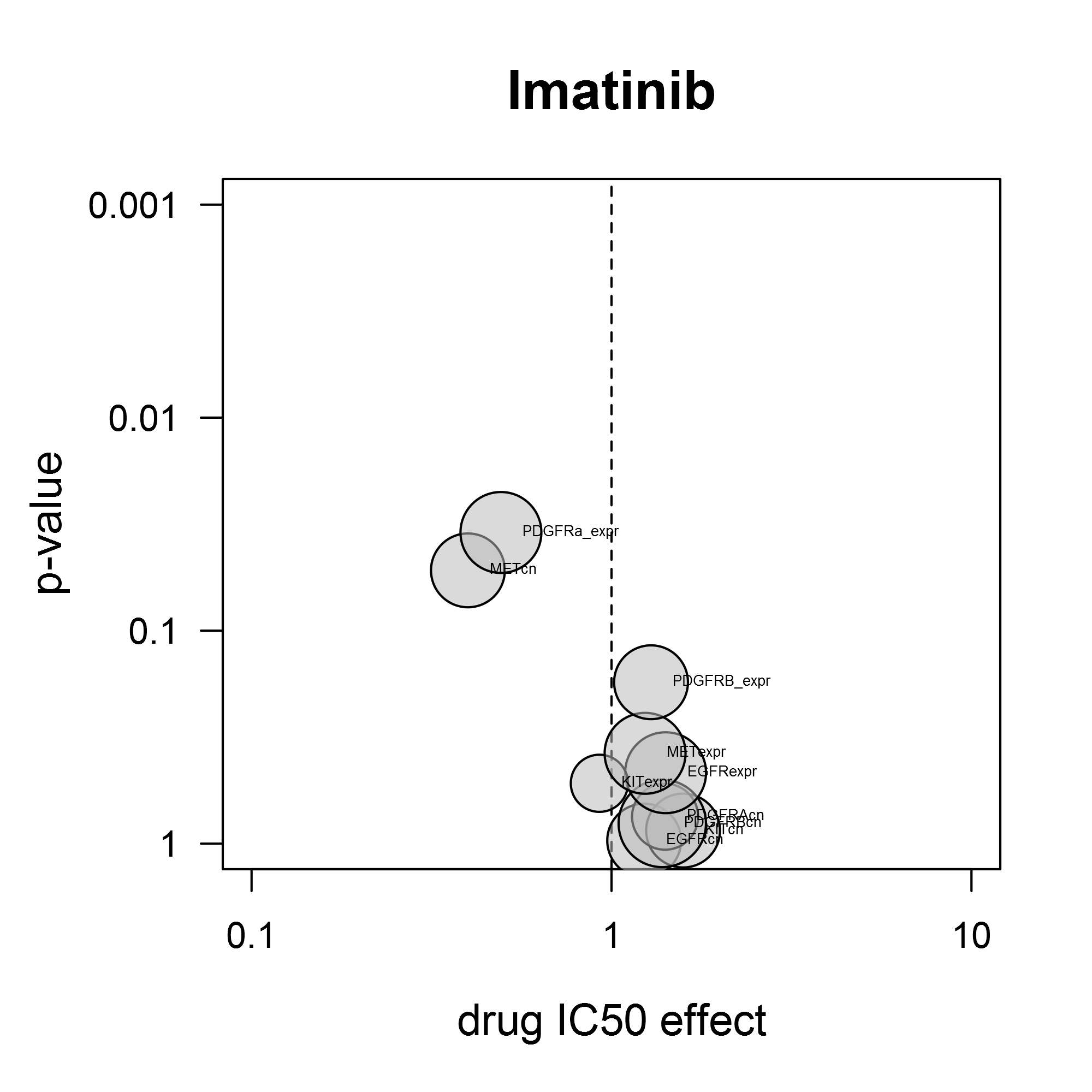


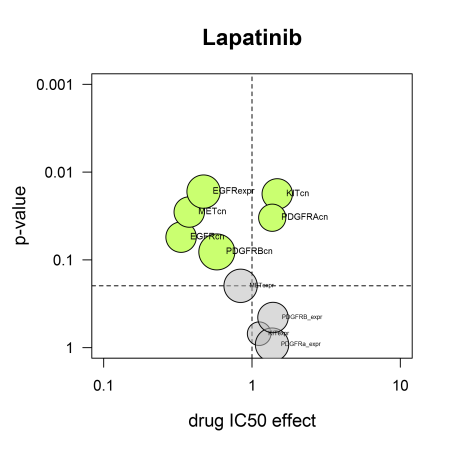

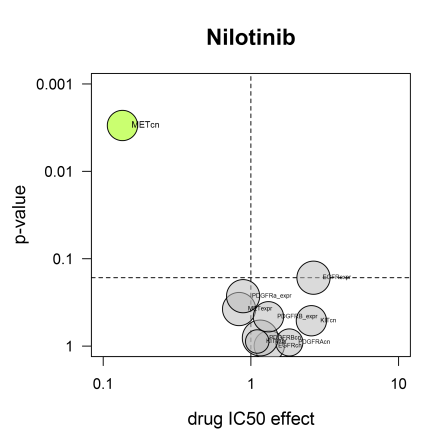

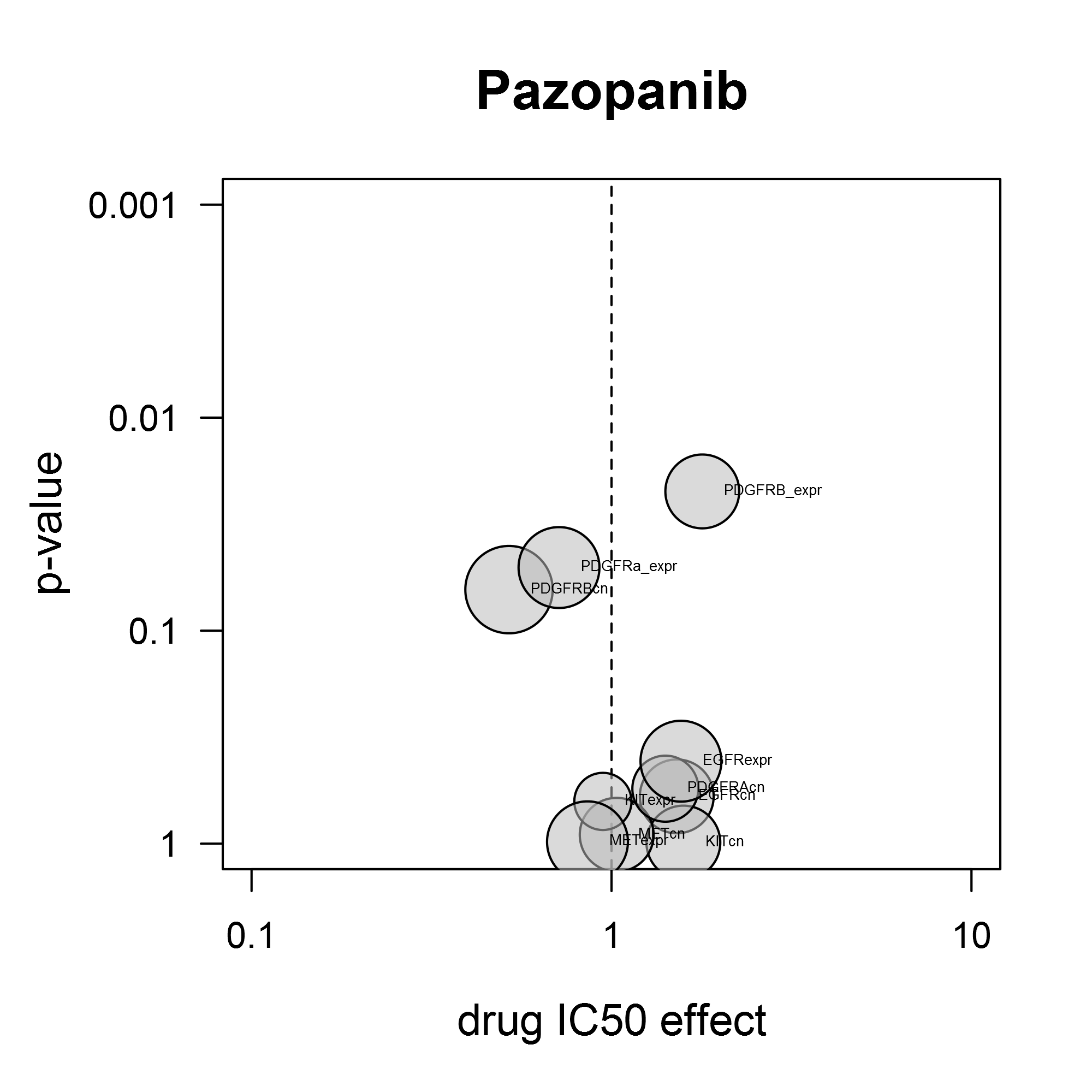


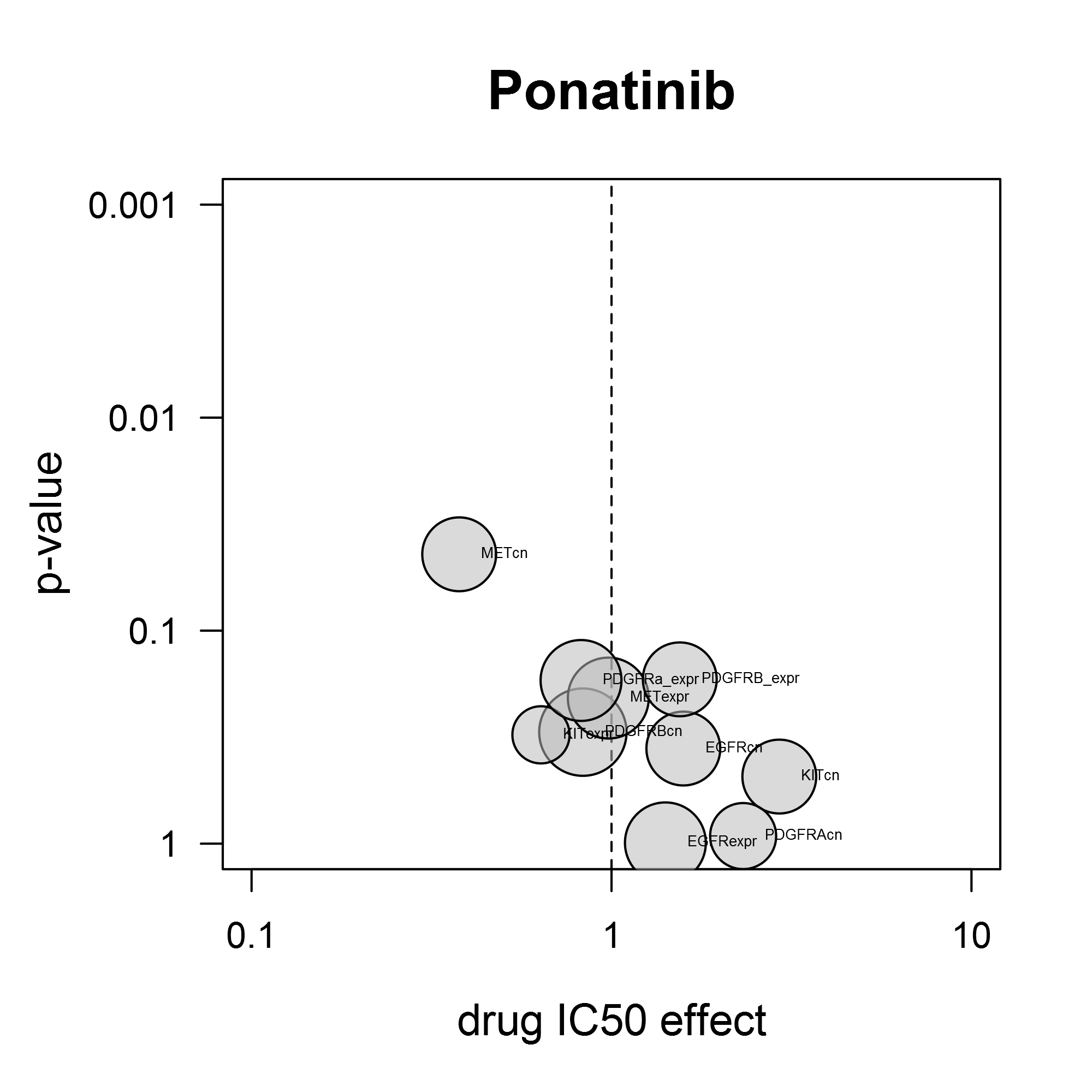

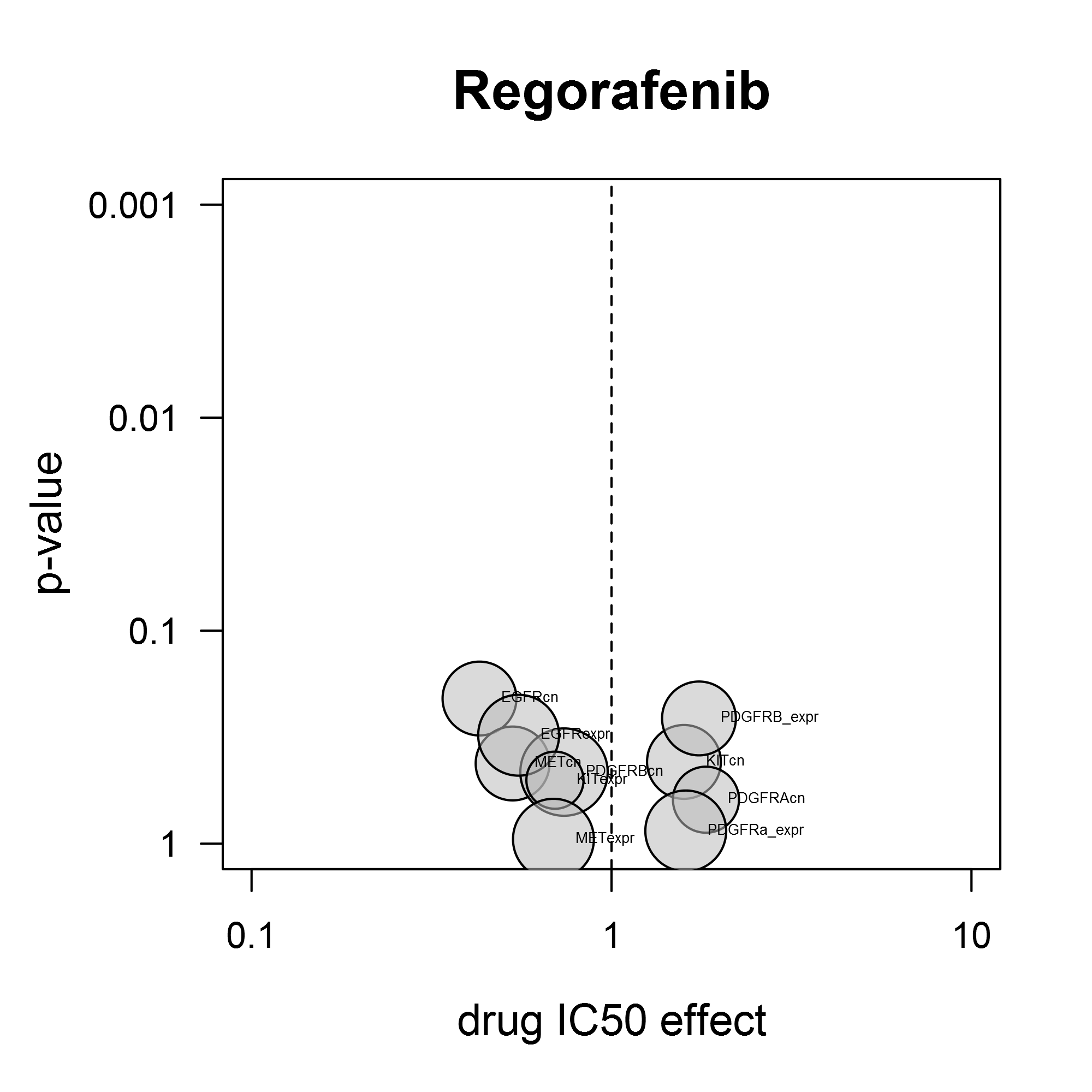

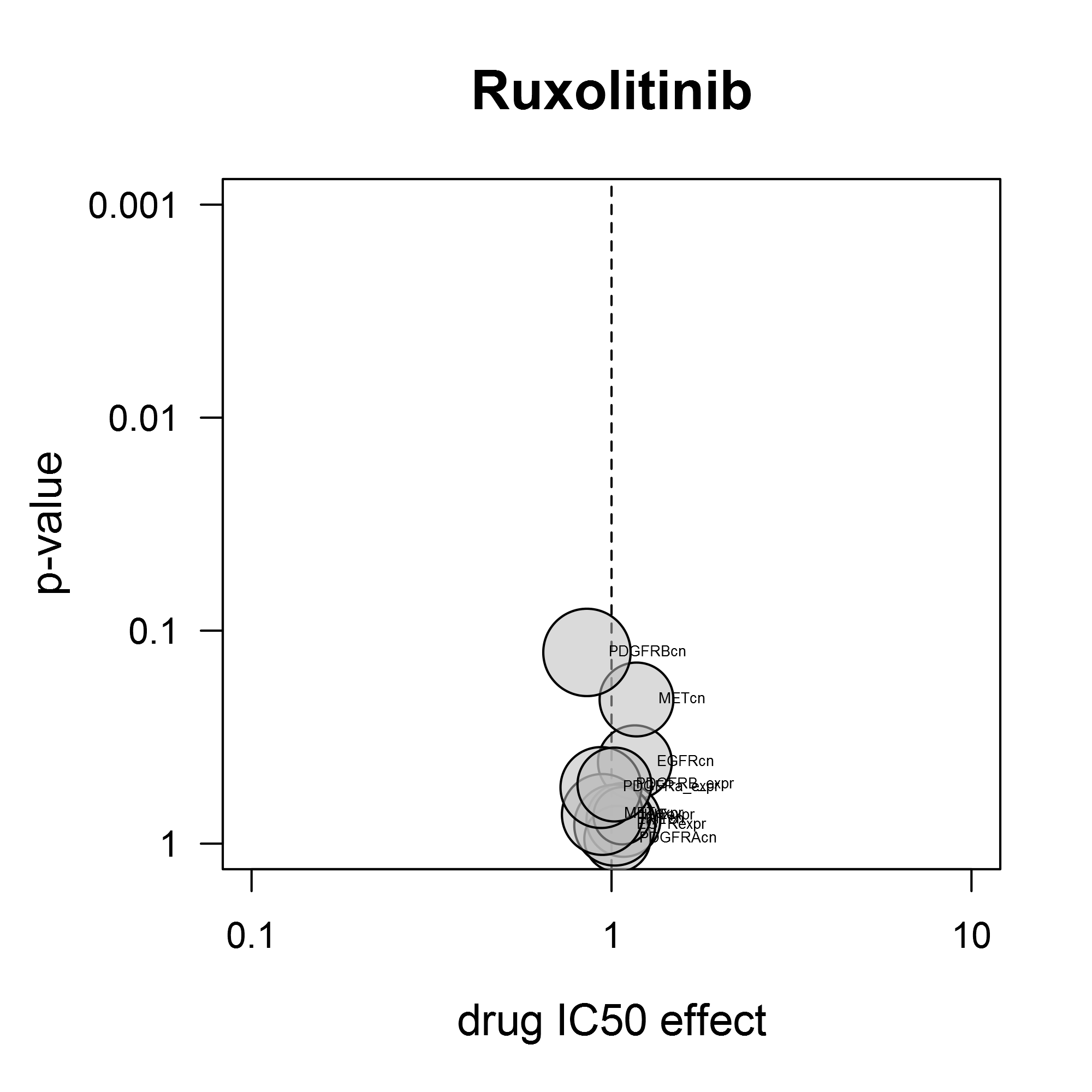


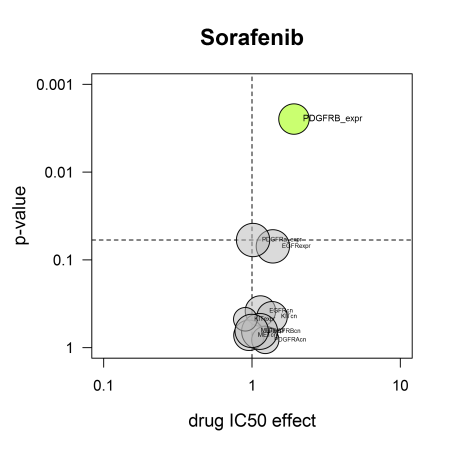

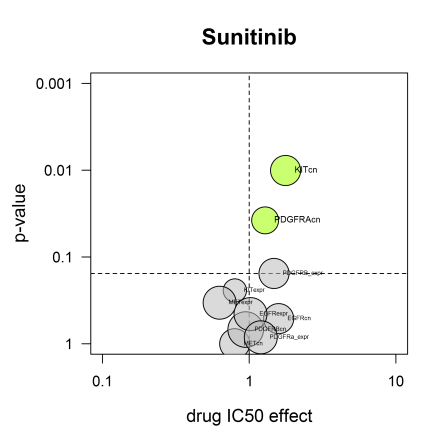

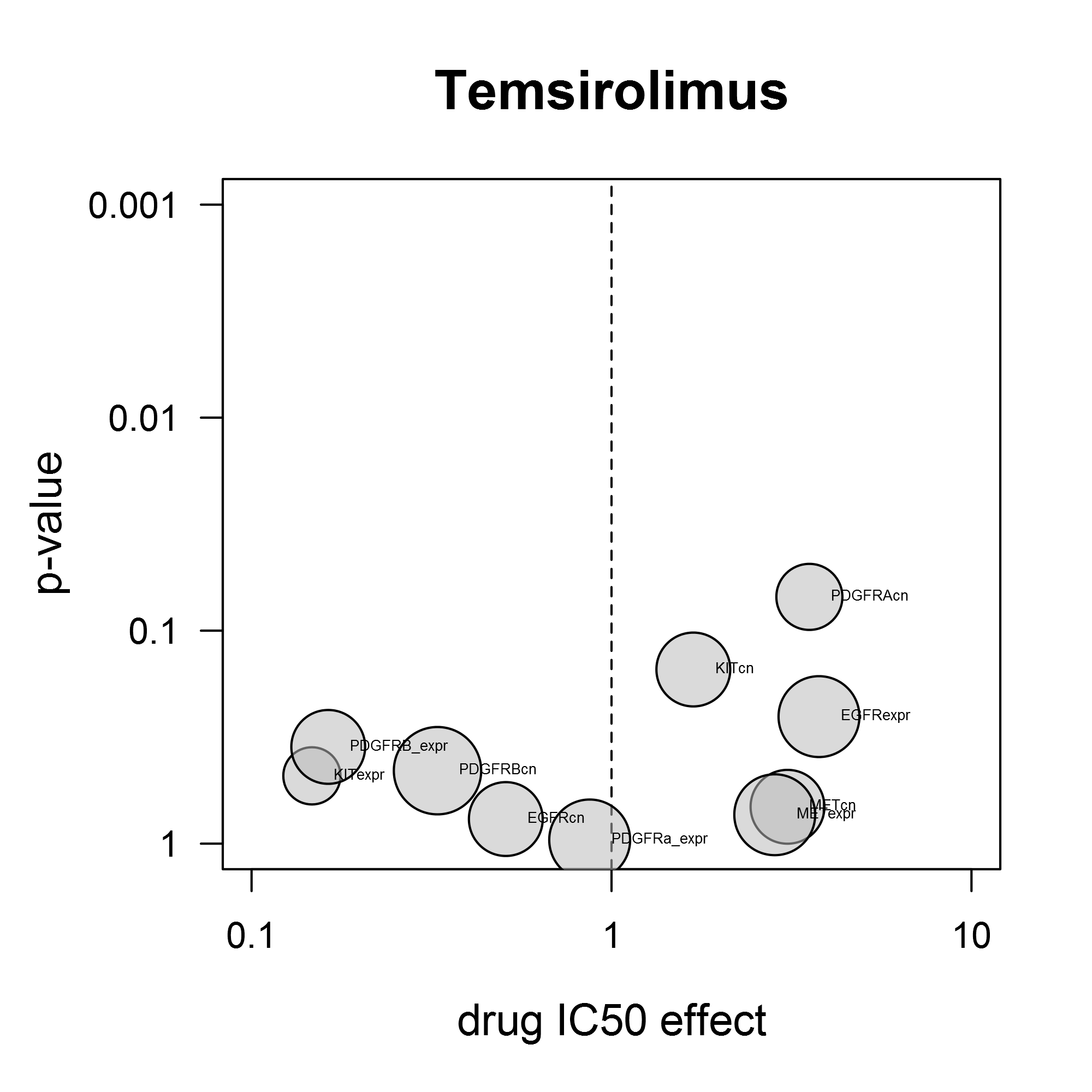


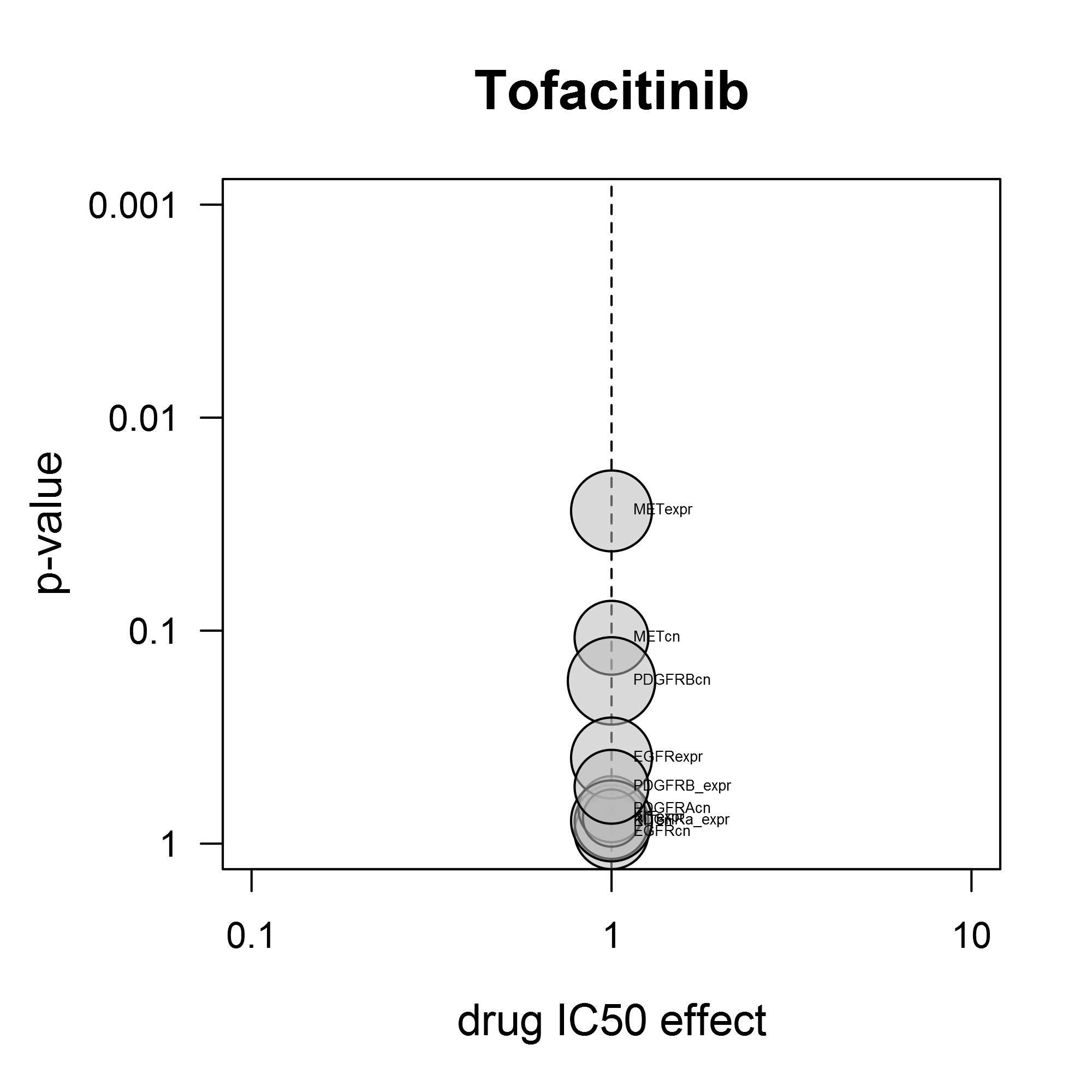

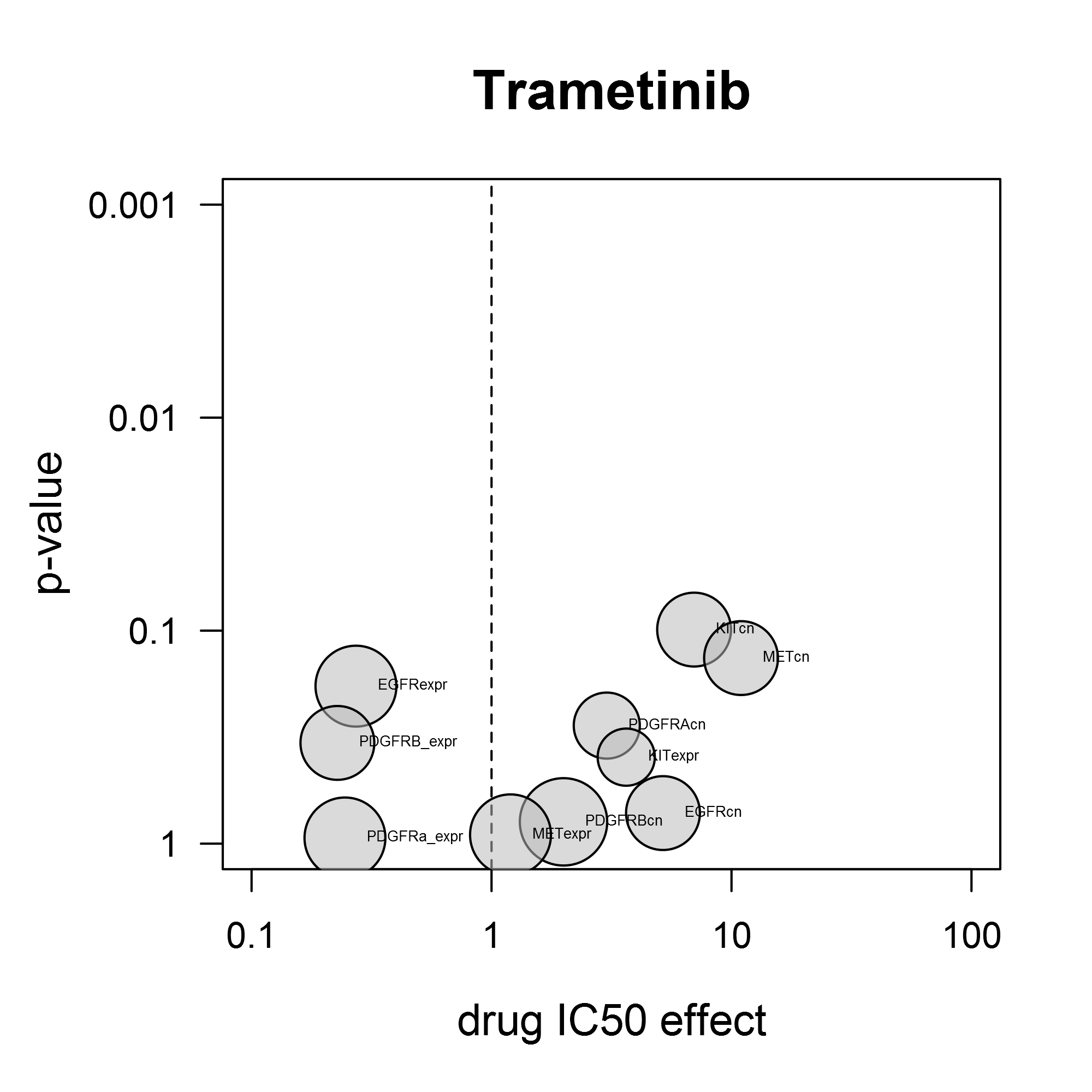

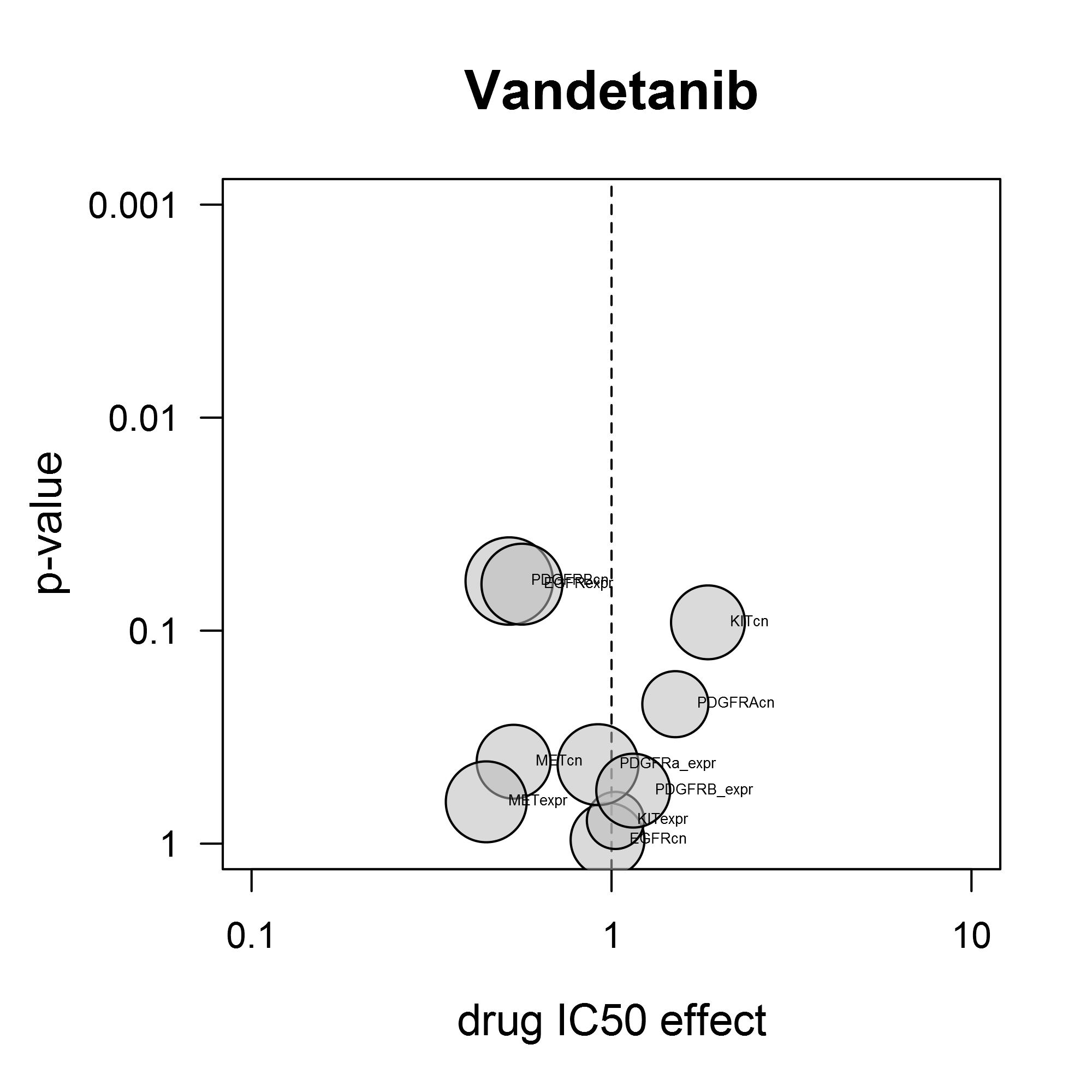


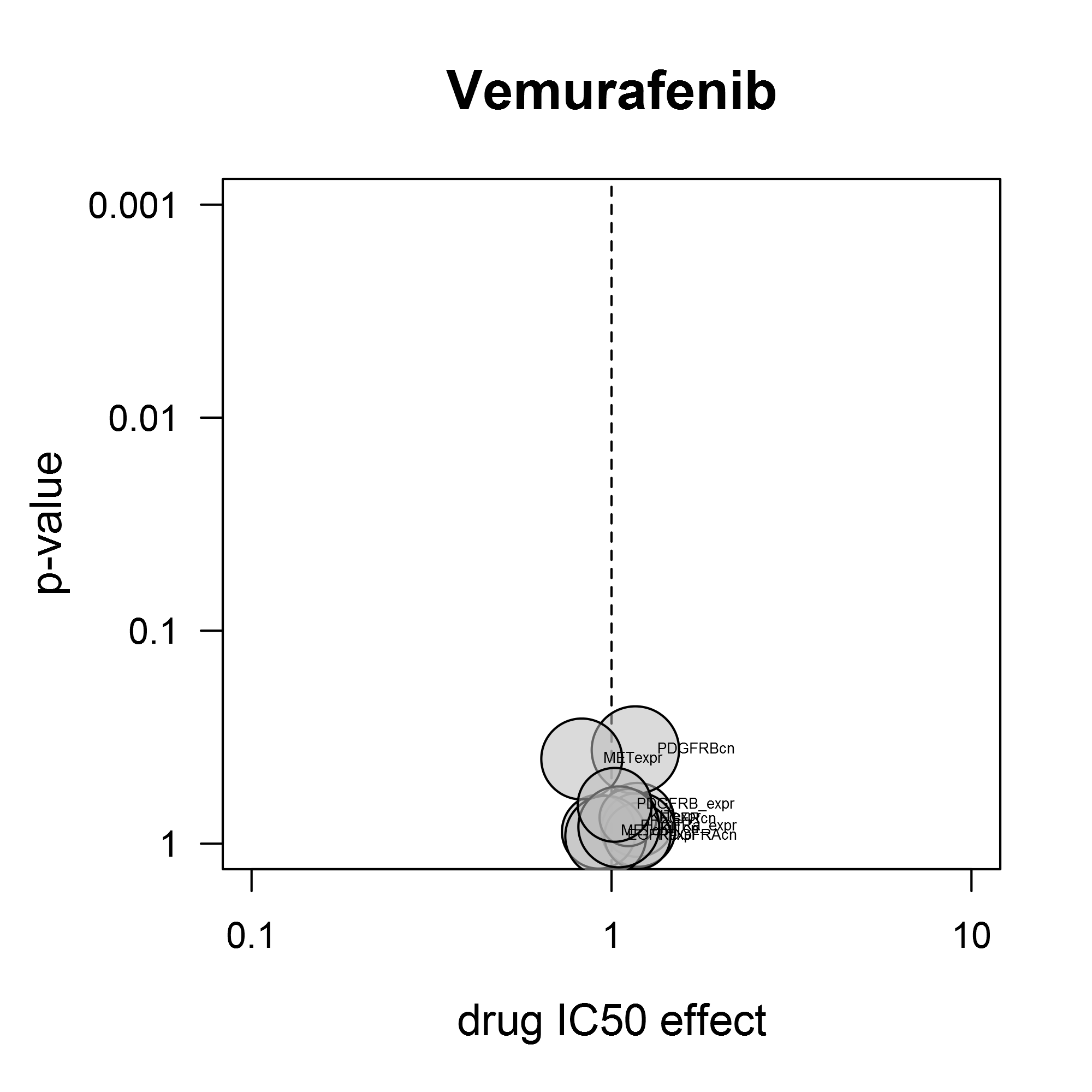


**Cytostatic therapies**

(doxorubicin 1-3 are a triplicate experiment to show the level of variation)


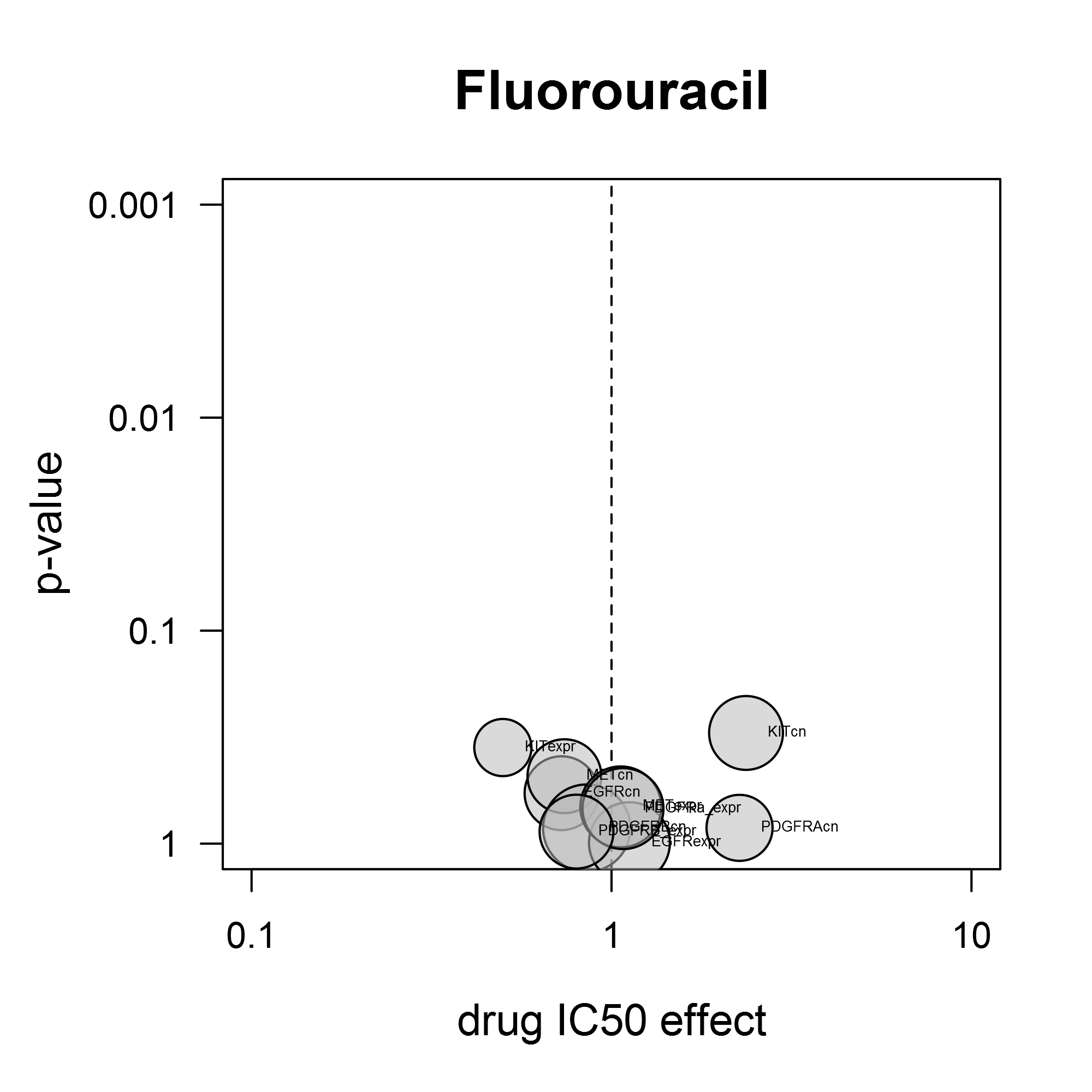

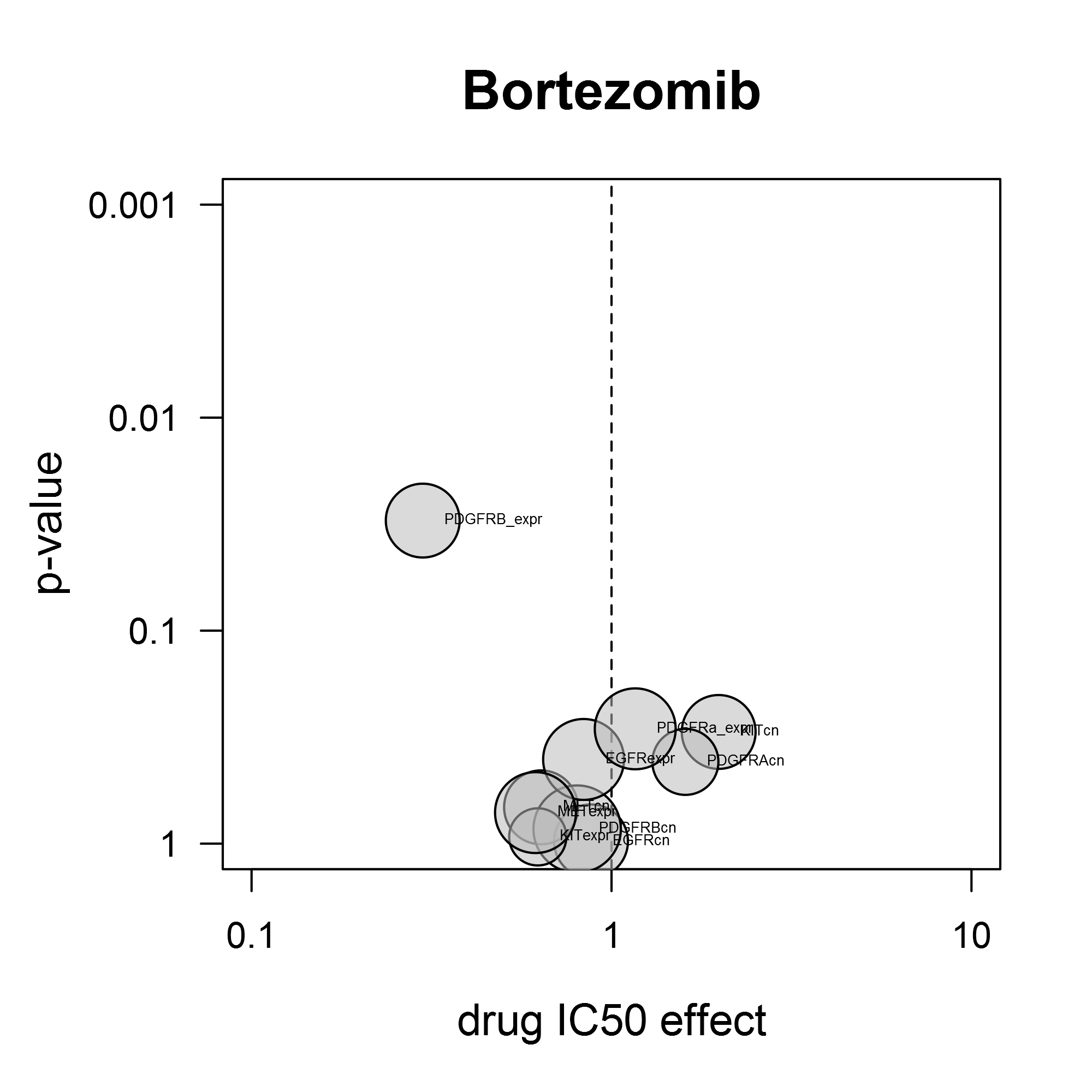

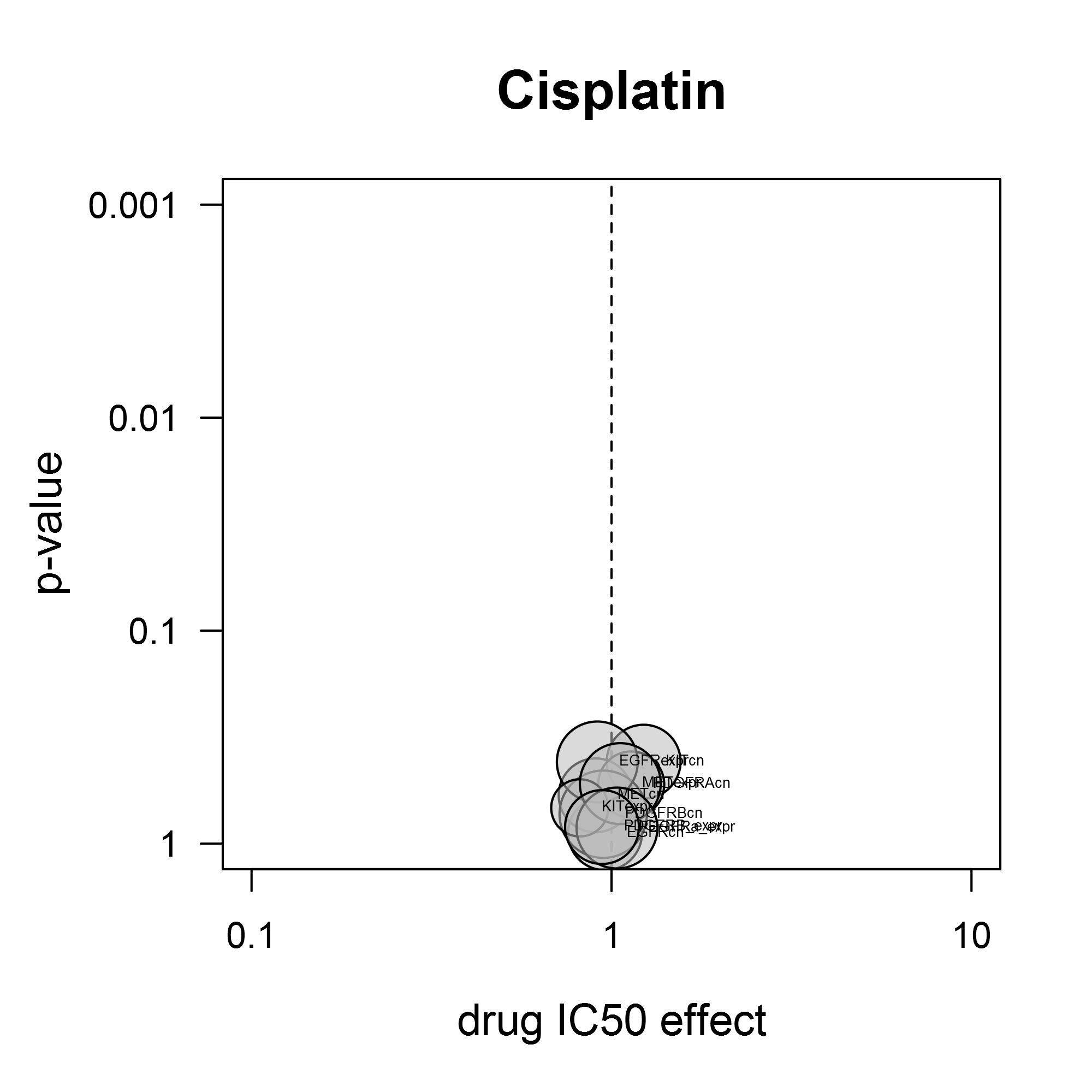


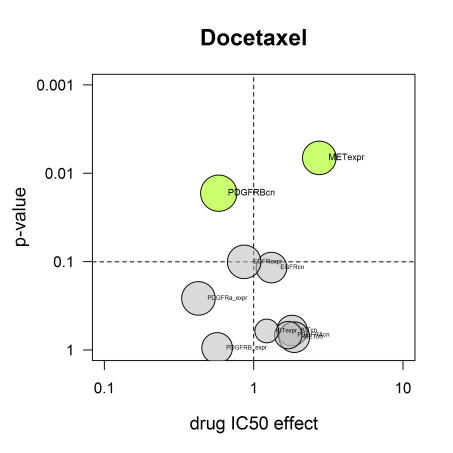

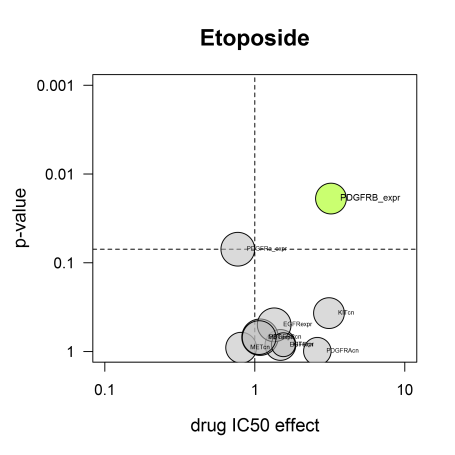

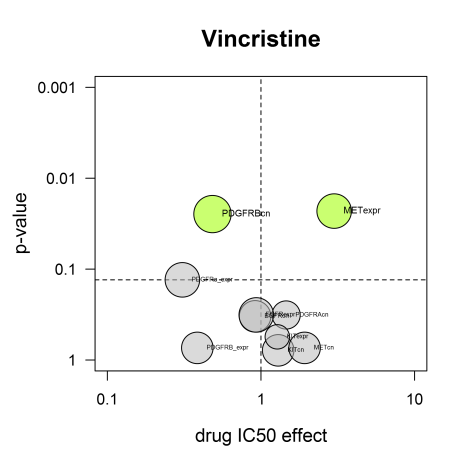


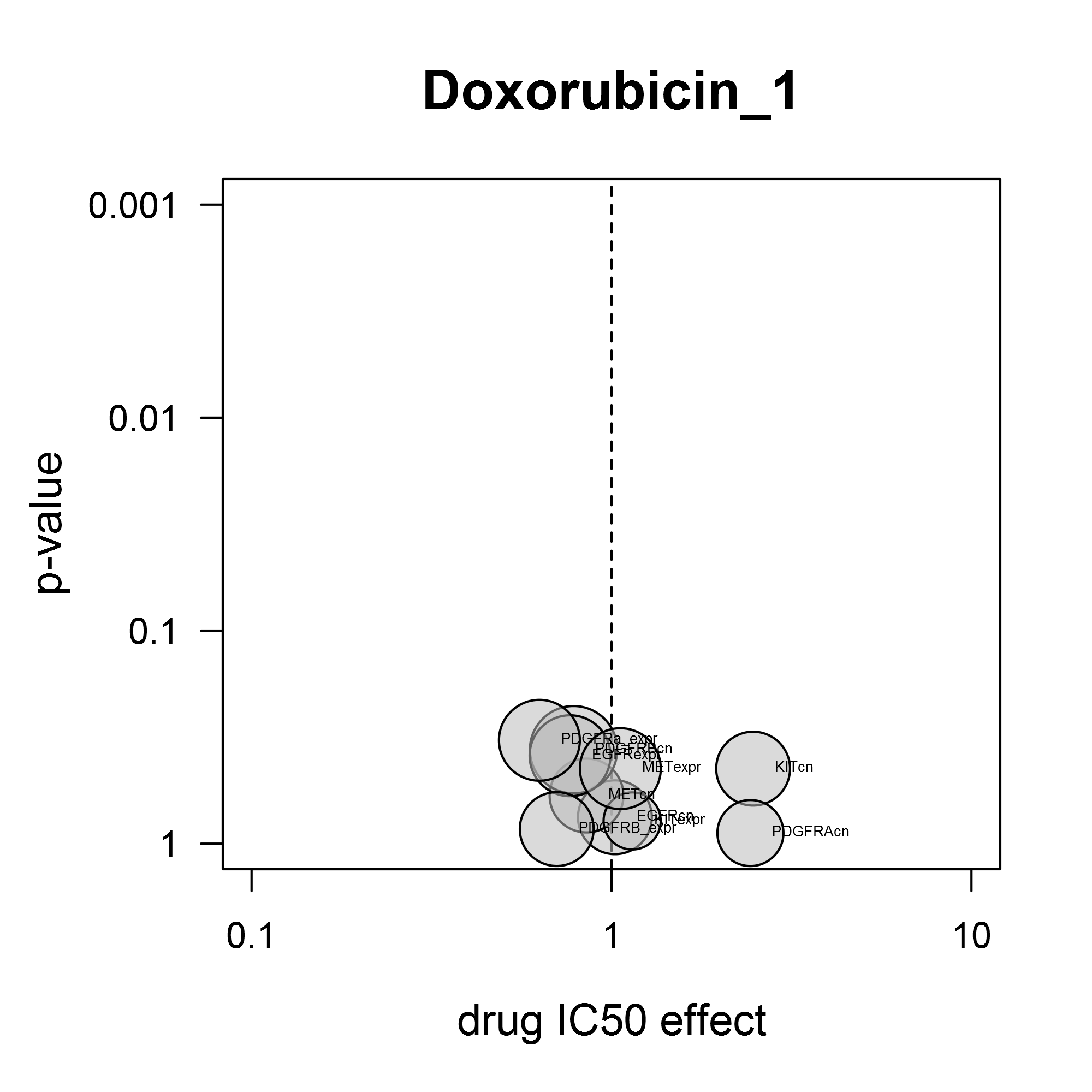

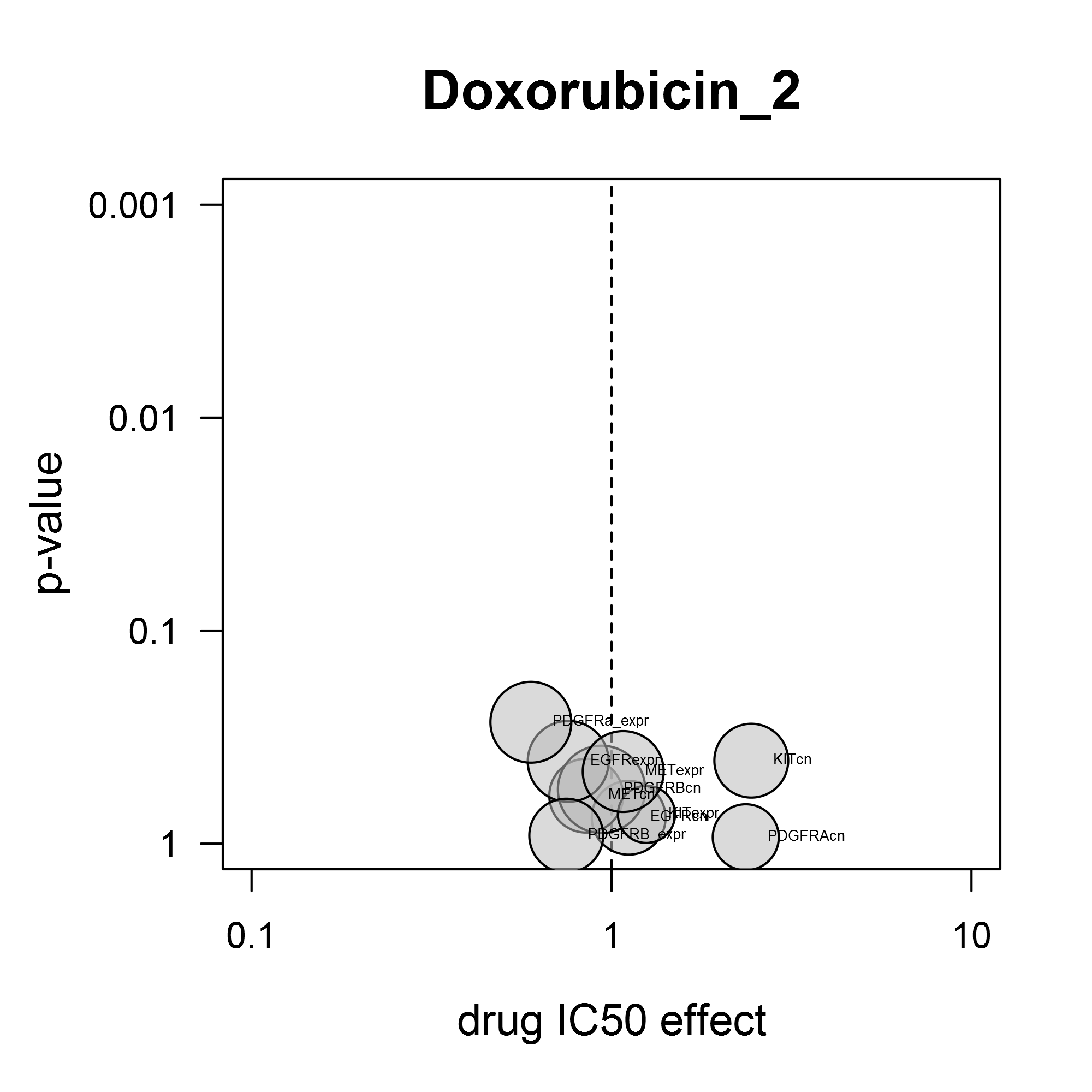

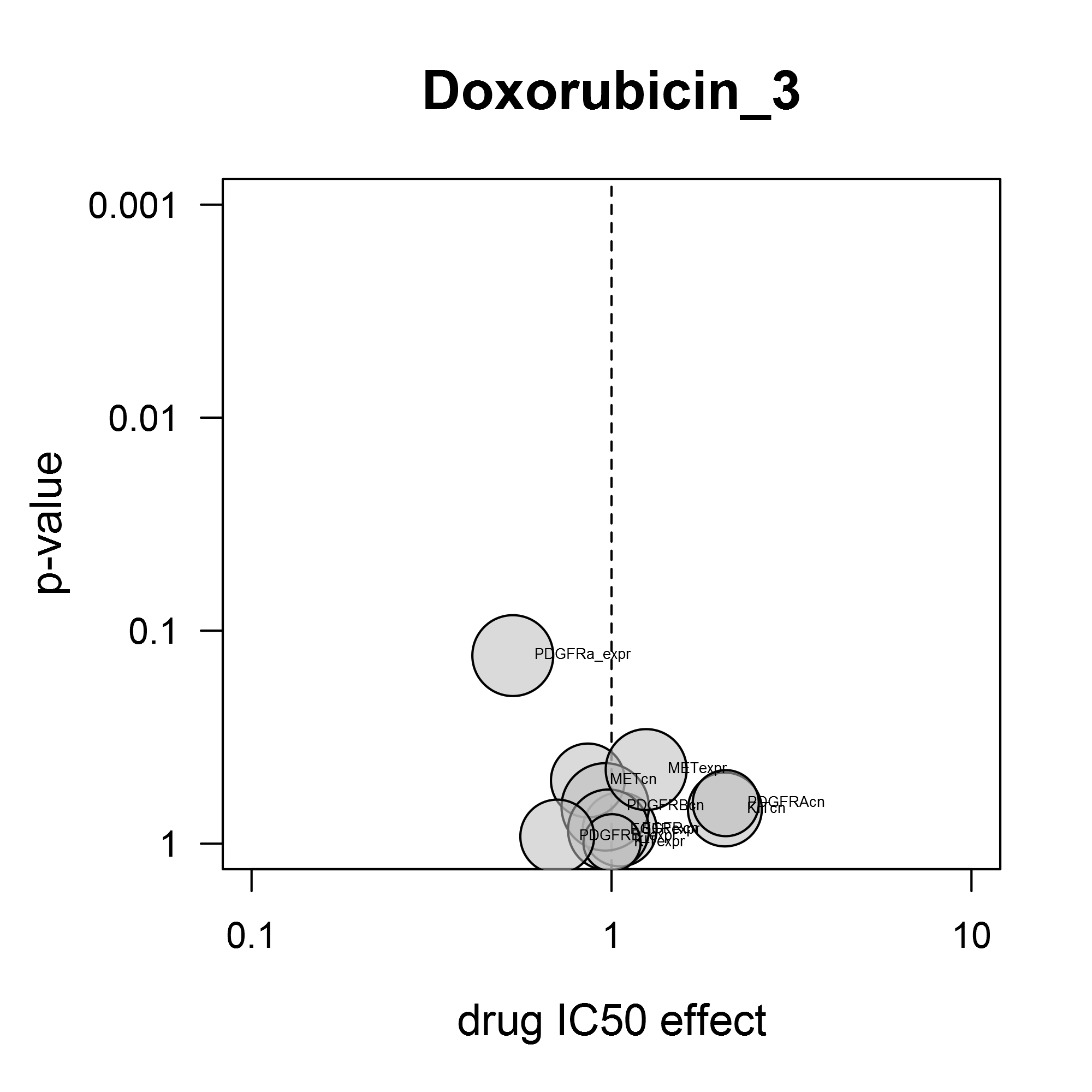

Supplement: Figure S5 — Volcano-analysis of drug sensitivity of twenty-five approved kinase inhibitors and seven cytostatic therapies to genetic changes in growth factor signalling. (DOCX) [file pone.0092146.s005.docx]
