# Supplementary material for: Comparison of the Cancer Gene Targeting and Biochemical Selectivities of All Targeted Kinase Inhibitors Approved for Clinical Use
Source: PLoS One. 2014 Mar 20;9(3):e92146. doi: 10.1371/journal.pone.0092146 (PMC3961306; doi:10.1371/journal.pone.0092146)
Supplement: Figure S6 — Volcano-analysis of the MEK inhibitors PD-0325901 and selumetinib (AZD-6244). (DOCX) [file pone.0092146.s006.docx]

Uitdehaag *et al*. supplementary Figure S6

**A
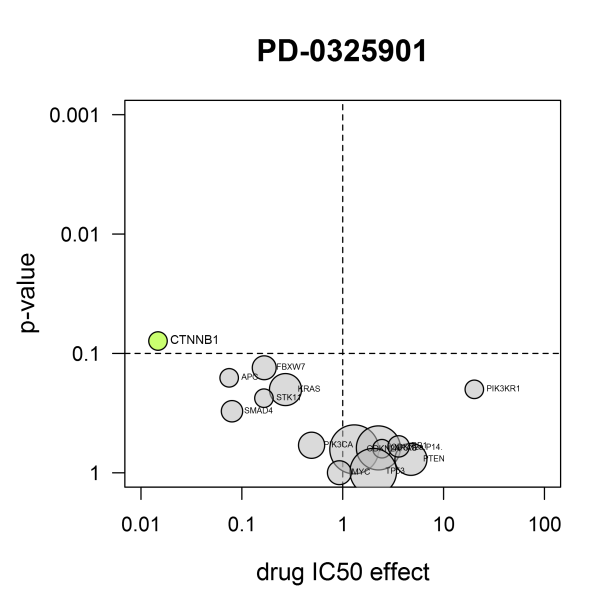
**

**B
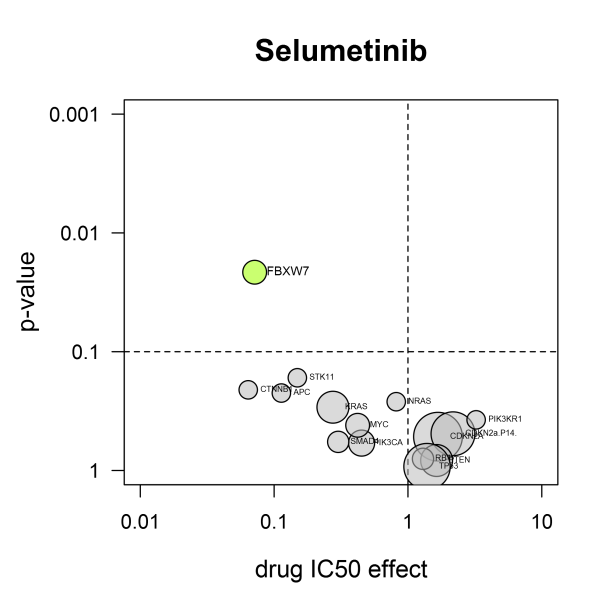
**

**Figure S6.** **Volcano-analysis of the MEK inhibitors PD-0325901 and selumetinib (AZD-6244).** A: Similar to trametinib, PD-0325901 is more active in cell lines harbouring a CTNNB1 (β-catenin) mutation. B: Also for selumetinib, presence of a CTNNB1 mutation sensitizes cell lines more than tenfold, although significance doesn’t rise above the p-value cutoff level (green arrow). To bring out the effects clearly, cancer genes were analysed that occur at least three times in the panel, and the significance cutoff for the Anova was set at a fixed level of 0.1.
