# Supplementary material for: Comparison of the Cancer Gene Targeting and Biochemical Selectivities of All Targeted Kinase Inhibitors Approved for Clinical Use
Source: PLoS One. 2014 Mar 20;9(3):e92146. doi: 10.1371/journal.pone.0092146 (PMC3961306; doi:10.1371/journal.pone.0092146)
Supplement: Figure S7 — Volcano-analysis of the EGFR inhibitors pelitinib (EKB-569) and neratinib (HKI-272). (DOCX) [file pone.0092146.s007.docx]

Uitdehaag *et al*. supplementary data

**A
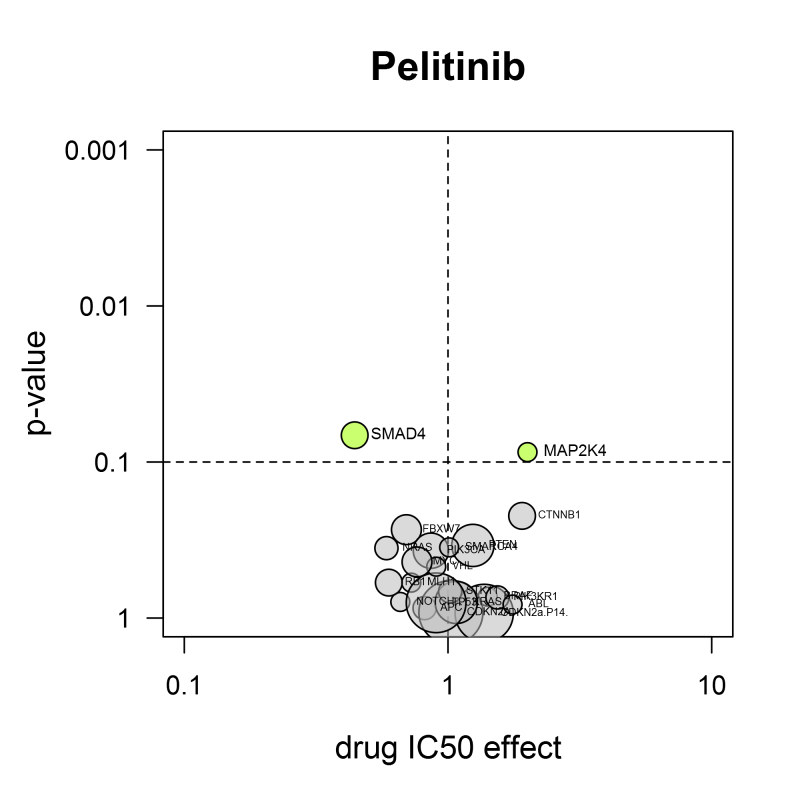
**

**B**
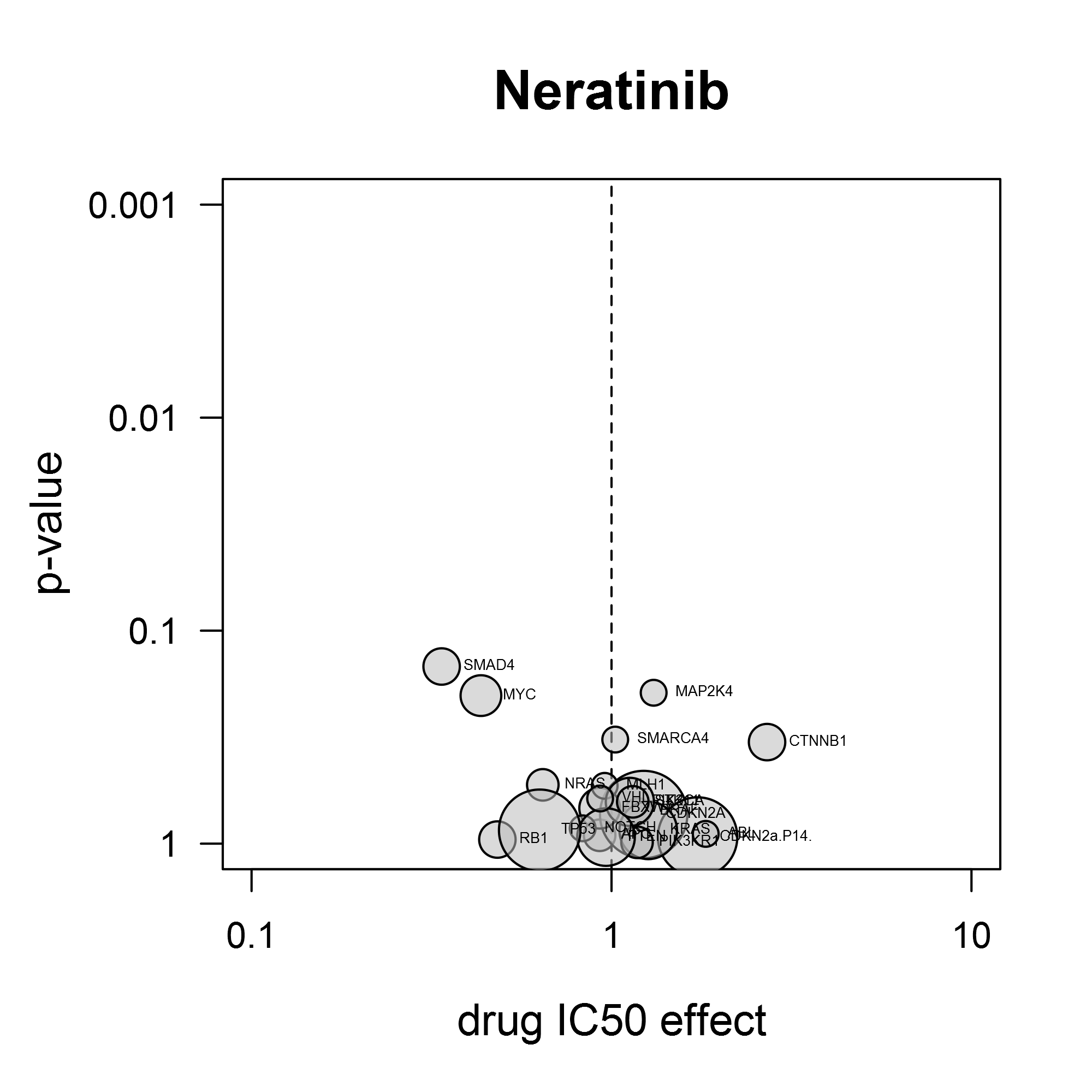


**Figure S7**. **Volcano-analysis of the EGFR inhibitors pelitinib (EKB-569) and neratinib (HKI-272).** A: Pelitinib is more active in cell lines containing SMAD4 mutations. B: Also for neratinib, presence of a SMAD4 mutation sensitizes cell lines, although significance doesn’t rise above the p-value cutoff level (green arrow). This confirms the findings with afatinib, another EGFR inhibitor, as described in the main text and erlotinib and gefitinib (Figure S4). The significance cutoff (dotted line) in these Anovas was set at a fixed level of 0.1.
