# Supplementary material for: Comparison of the Cancer Gene Targeting and Biochemical Selectivities of All Targeted Kinase Inhibitors Approved for Clinical Use
Source: PLoS One. 2014 Mar 20;9(3):e92146. doi: 10.1371/journal.pone.0092146 (PMC3961306; doi:10.1371/journal.pone.0092146)
Supplement: Figure S8 — Selectivity entropy (Ssel) can be estimated on basis of single concentration data. (DOCX) [file pone.0092146.s008.docx]

Uitdehaag *et al*. supplementary Figure S8

**Figure S8**. **Selectivity entropy (S_sel_) can be estimated on basis of single concentration data.** ‘True’ S_sel_ values (x-axis) were calculated using established procedures [33] on basis of IC_50_ profiles of nine kinase inhibitors [11]. Estimated S_sel_ values (y-axis) were calculated by taking %-inhibition data (*%I*) at 1 μM inhibitor concentration of the same inhibitors [11] and transforming these to IC_50_s with the formula IC_50_ (μM) = ((100/*%I*)-1). This assumes that dose-response curves for all kinase-inhibitor pairs start at 0 % inhibition and end at 100 % inhibition with a Hill-coefficient of 1 and that the % inhibition is without error [54]. Estimated IC_50_s were restricted to values between 1.0 nM and 999 μM. The correlation between the ‘true’ and the estimated values shows that estimates are useful to get an approximation of a compound’s selectivity-entropy.
