# Supplementary material for: Comparison of the Cancer Gene Targeting and Biochemical Selectivities of All Targeted Kinase Inhibitors Approved for Clinical Use
Source: PLoS One. 2014 Mar 20;9(3):e92146. doi: 10.1371/journal.pone.0092146 (PMC3961306; doi:10.1371/journal.pone.0092146)
Supplement: Table S1 — Names and sources of approved kinase inhibitor drugs used in this study. (DOCX) [file pone.0092146.s009.docx]

Uitdehaag *et al*. supplementary Table S1

| Inhibitor name | Marketed name | | Compound code | Drug manufacturer | Compound  supplier |
| --- | --- | --- | --- | --- | --- |
| Imatinib | Gleevec® | | STI-571 | Novartis | LC labs |
| Dasatinib | Sprycel® | | BMS-354825 | Bristol Myers Squibb | LC labs |
| Nilotinib | Tasigna® | | AMN-107 | Novartis | Axon MedChem |
| Ponatinib | Iclusig® | | AP-24534 | Ariad | Selleck Chemicals |
| Bosutinib | Bosulif® | | SKI-606 | Wyeth / Pfizer | Selleck Chemicals |
| Vemurafenib | Zelboraf® | | PLX-4032 | Roche | Selleck Chemicals |
| Dabrafenib | Tafinlar® | | GSK-2118436 | Glaxo Smithkline | Selleck Chemicals |
| Erlotinib | Tarceva® | | OSI-774 | Genentech / Roche | LC labs |
| Gefitinib | Iressa® | | ZD-1839 | Astra Zeneca | LC labs |
| Lapatinib | Tykerb® | | GW-2016 | Glaxo Smithkline | LC labs |
| Afatinib | Gilotrif® | | BIBW-2992 | Boehringer Ingelheim | Selleck Chemicals |
| Temsirolimus | Torisel® | | CCI-779 | Wyeth / Pfizer | Selleck Chemicals |
| Everolimus | Afinitor® | | RAD-001 | Novartis | Selleck Chemicals |
| Tofacitinib | Xeljanz® | | CP-690550 | Pfizer | Axon MedChem |
| Ruxolitinib | Jakafi® | | INCB-18424 | Incyte | Selleck Chemicals |
| Vandetanib | Caprelsa® | | ZD-6474 | Astra Zeneca | Selleck Chemicals |
| Pazopanib | Votrient® | | GW-786034 | Glaxo Smithkline | Selleck Chemicals |
| Sunitinib | Sutent® | | SU-11248 | Sugen / Pfizer | LC labs |
| Sorafenib | Nexavar® | | BAY-439006 | Onyx / Bayer | LC labs |
| Axitinib | Inlyta® | | AG-013736 | Pfizer | Selleck Chemicals |
| Regorafenib | Stivarga® | | BAY73-4506 | Bayer | Selleck Chemicals |
| Cabozantinib | Cometriq® | | XL-184 | Exelixis | Selleck Chemicals |
| Crizotinib | Xalkori® | | PF-02341066 | Pfizer | Selleck Chemicals |
| Trametinib | Mekinist® | | JTP-74057 | Glaxo Smithkline | Selleck Chemicals |
| Fasudil | Eril® | | HA-1077 | Eisai | Selleck Chemicals |
|  | |  |  |  |  |

**Table S1**. **Names and structures of approved kinase inhibitor drugs.** ‘Drug manufacturer’ indicates the company marketing the drug. ‘Compound supplier’ indicates the supplier of the compound as research tool for this study.
