# Supplementary material for: Comparison of the Cancer Gene Targeting and Biochemical Selectivities of All Targeted Kinase Inhibitors Approved for Clinical Use
Source: PLoS One. 2014 Mar 20;9(3):e92146. doi: 10.1371/journal.pone.0092146 (PMC3961306; doi:10.1371/journal.pone.0092146)
Supplement: Table S2 — Frequency of point mutations in validated cancer genes in the cell panel. (DOCX) [file pone.0092146.s010.docx]

Uitdehaag *et al*. supplementary Table S2

| Rank | Gene | Oncolines^TM^ mutations | Oncolines^TM^ % | CCL mutations | CCL % |
| --- | --- | --- | --- | --- | --- |
| 1 | TP53 | 20 | *13.8* | 495 | *25.7* |
| 2 | CDKN2A | 23 | *15.9* | 293 | *15.2* |
| 3 | CDKN2a(P14) | 19 | *13.1* | 252 | *13.1* |
| 4 | RB1 | 4 | *2.8* | 107 | *5.6* |
| 5 | PTEN | 10 | *6.9* | 104 | *5.4* |
| 6 | KRAS | 10 | *6.9* | 89 | *4.6* |
| 7 | BRAF | 2 | *1.4* | 66 | *3.4* |
| 8 | PIK3CA | 7 | *4.8* | 64 | *3.3* |
| 9 | NRAS | 3 | *2.1* | 48 | *2.5* |
| 10 | APC | 3 | *2.1* | 35 | *1.8* |
| 11 | STK11 | 3 | *2.1* | 32 | *1.7* |
| 12 | SMAD4 | 4 | *2.8* | 31 | *1.6* |
| 13 | FBXW7 | 5 | *3.4* | 29 | *1.5* |
| 14 | KDM6A | 1 | *0.7* | 28 | *1.5* |
| 15 | NF1 | 1 | *0.7* | 24 | *1.2* |
| 16 | NF2 | 1 | *0.7* | 23 | *1.2* |
| 17 | CTNNB1 | 4 | *2.8* | 17 | *0.9* |
| 18 | MAP2K4 | 2 | *1.4* | 16 | *0.8* |
| 19 | NOTCH | 2 | *1.4* | 16 | *0.8* |
| 20 | SMARCA | 2 | *1.4* | 14 | *0.7* |
| Total, top 20 | | 126 | *86.9* | 1783 | *92.5* |
| Other genes | | 19 | *13.1* | 144 | *7.5* |
| Total 48 genes | | 145 | *100.0* | 1927 | *100.0* |

**Table S2**. **Frequency of point mutations in validated cancer genes in the cell panel**. Shown are 20 out of 48 validated cancer genes as documented in the capillary sequencing CCL dataset [4]. The total of these 20 mutations captures more than 90 % of the total mutations in the CCL cell line project.
