# Supplementary material for: Comparison of the Cancer Gene Targeting and Biochemical Selectivities of All Targeted Kinase Inhibitors Approved for Clinical Use
Source: PLoS One. 2014 Mar 20;9(3):e92146. doi: 10.1371/journal.pone.0092146 (PMC3961306; doi:10.1371/journal.pone.0092146)
Supplement: Table S3 — List of genes mutated in the Oncolines panel that have been used to investigate drug sensitivity. (DOCX) [file pone.0092146.s011.docx]

Uitdehaag *et al*. supplementary Table S3

| COSMIC (CCL) data | no. cell lines | CCLE data | no. cell lines |
| --- | --- | --- | --- |
| Point mutations |  | **High copy number** |  |
| APC | 3 | EGFR | 5 |
| BRAF | 2 | KIT | 5 |
| CDKN2A | 23 | MET | 5 |
| CDKN2a(P14) | 19 | PDGFRα | 4 |
| CTNNB1 | 4 | PDGFRβ | 7 |
| FBXW7 | 5 |  |  |
| KRAS | 10 | **mRNA overexpression** | |
| MAP2K4 | 2 | EGFR | 6 |
| MLH1 | 2 | KIT | 3 |
| MSH2 | 2 | MET | 6 |
| NOTCH | 2 | PDGFRα | 6 |
| NRAS | 3 | PDGFRβ | 5 |
| PIK3CA | 7 |  |  |
| PIK3KR1 | 3 |  |  |
| PTEN | 10 |  |  |
| RB1 | 4 |  |  |
| SMAD4 | 4 |  |  |
| SMARCA4 | 2 |  |  |
| STK11 | 3 |  |  |
| TP53 | 20 |  |  |
| VHL | 2 |  |  |
| High copy number | |  |  |
| MYC | 5 |  |  |
| BCRABL | 2 |  |  |

**Table S3.** **List of genes mutated in the Oncolines panel that have been used to investigate drug sensitivity**. Left column: genes that are most commonly genetically changed in cancer, as documented in the CCL database [4]. Right column: five relevant growth factor kinases that are often involved in cancerous growth. Expression and copy number data were obtained from the CCLE database [5]. Numbers behind each gene refer to the number of cell lines in the 44-cell lines Oncolines panel harbouring genetic changes in that particular target.
