# Supplementary material for: Comparison of the Cancer Gene Targeting and Biochemical Selectivities of All Targeted Kinase Inhibitors Approved for Clinical Use
Source: PLoS One. 2014 Mar 20;9(3):e92146. doi: 10.1371/journal.pone.0092146 (PMC3961306; doi:10.1371/journal.pone.0092146)
Supplement: Table S4 — Statistical power analysis to determine cutoff levels for significance in the cell line panel. (DOCX) [file pone.0092146.s012.docx]

Uitdehaag *et al*. supplementary Table S4

| Sensitive | Insensitive | Min effect size | Min IC_50_ shift |
| --- | --- | --- | --- |
| 2 | 42 | 2.07 | 9.9 |
| 3 | 41 | 1.71 | 6.7 |
| 4 | 40 | 1.50 | 5.3 |
| 5 | 39 | 1.36 | 4.5 |
| 10 | 34 | 1.03 | 3.1 |
| 22 | 22 | 0.86 | 2.6 |

**Table S4**. **Statistical power analysis to determine significant effects in the cell line panel.** (MacCallum *et al*, Psychological Methods 1, 130-149 (1996)). Given a certain number of sensitive and insensitive cell lines, the analysis gives the minimal factor shift in drug response IC_50_ needed for a significant effect (p < 0.05). Numbers are based on a two-tailed t-test with two samples and a power of 0.8 and were calculated in R. Effect size is defined as the difference between the sample averages (in ^10^logIC_50_), divided by their standard deviation. Effect size was converted to IC_50_ shift by using the standard deviation of all ^10^logIC_50_s of doxorubicin replicates across all cell lines in the panel. This value for the standard deviation (0.48) is an overestimation as it incorporates biological differences in doxorubicin response across different cell lines.
