# Supplementary material for: Comparison of the Cancer Gene Targeting and Biochemical Selectivities of All Targeted Kinase Inhibitors Approved for Clinical Use
Source: PLoS One. 2014 Mar 20;9(3):e92146. doi: 10.1371/journal.pone.0092146 (PMC3961306; doi:10.1371/journal.pone.0092146)
Supplement: Table S7 — List of all cell lines in the Oncolines panel. (DOCX) [file pone.0092146.s015.docx]

Uitdehaag *et al*. supplementary Table S7

**Table S7**. List of all cell lines in the Oncolines^TM^ 44 cell line panel
